# Selective Sorbent Design: CaS Aerogel for Rapid Remediation of Aqueous Pb (II)

**DOI:** 10.1021/acs.chemmater.5c03082

**Published:** 2026-03-30

**Authors:** Md. Masudur Rhaman, Stephanie L. Brock

**Affiliations:** Department of Chemistry, Wayne State University, 5101 Cass Avenue, Detroit, Michigan 48202, United States

## Abstract

Heavy metals are a persistent environmental problem due to their high toxicity, even at very low concentrations (parts per billion, ppb). The removal of such diluted heavy metals is challenging because of the competition the counterions (Ca^2+^, Na^+^, Mg^2+,^ etc.) present in natural water bodies. The design of sorbents capable of removing ions below the action limit (15 ppb for Pb^2+^) requires a strong driving force for selective uptake and rapid removal. In this work, we report the synthesis of porous CaS aerogels (surface area = 143.6 m^2^/g) by oxidative assembly of CaS nanoparticles and describe their use in selective Pb^2+^ ion remediation from water. Despite the presence of amorphous CaCO_3_ (up to 50 wt %) in the gel network, the gels demonstrated a capacity of 17.1 mmol Pb/g aerogel (3543 mg/g), and this could be augmented to 22.5 mmol Pb/g aerogel (4593 mg/g) by modifying the synthesis to reduce CaCO_3_ content to ca. 15 wt %. Moreover, the selectivity of CaS aerogels toward Pb^2+^ ions is high, as evidenced by little-to-no change in the distribution constant (*K*_d_ ∼ 10^4^) in the presence of competing ions (1 M) such as Na^+^, Mg^2+^, and Ca^2+^. During remediation with low concentrations (100 ppb) of Pb^2+^ with CaS aerogels, the level of Pb^2+^ dropped to 5.4 ppb (below the 15 ppb EPA limit) within 1 h with a 95.4% removal efficiency. In contrast to the CO_2_ supercritically dried aerogels, lower surface area ambient dried gels (xerogels) only remove 40% of the lead ions from a 100 ppb solution, saturating within 1 h. The efficiency and rapidity of selective Pb^2+^ uptake using CdS aerogels arise from a combination of a strong thermodynamic driving force for cation exchange (*K*_eq_ = 2.5 × 10^27^) and chemisorption along with favorable kinetics associated with the high surface area porous architecture. These results show that formation of high surface area metal chalcogenide aerogels by oxidative assembly to form nanocrystalline architectures, as previously demonstrated for II–VI and IV–VI semiconductors, can be extended to the more highly ionic alkaline earth sulfides.

## Graphical abstract

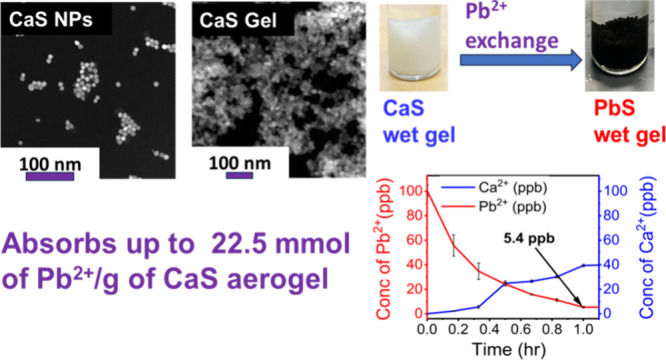

## Introduction

Heavy metal contamination of water bodies is a consequence of waste discharge associated with industrialization.^1^ Heavy metals, defined as having high atomic weight and density greater than 5 g/cm^3^, include As, Pb, Cd, and Hg; they are known carcinogens and cause damage to the nervous system, immune system, kidney, and bones if present in drinking water above safe limits.^2^ Therefore, the removal of heavy metals from wastewater and drinking water is imperative. Conventional removal techniques, including precipitation, coagulation, and adsorption, often suffer from low capacity and selectivity.^3^ In contrast, ion exchange is a process that enables the selective removal of ions of interest with the potential for high capacity.^4^ However, standard ion-exchange materials are based on oxide frameworks (i.e., inorganic clays^5,6^ and zeolites^7–9^ ), which are categorized as hard Lewis bases and therefore have a poor affinity to heavy metal ions such as Pb^2+^, which are soft Lewis acids, leading to low removal capacity (ca. 1 mmol Pb^2+^/g) and selectivity. To overcome these drawbacks, researchers have modified the surface of oxidic materials by functionalizing with thiol groups (soft Lewis bases), enhancing removal capacity up to 2 mmol Pb^2+^/g.^10–13^

Metal chalcogenides are ideal candidates for remediation because of the presence of soft Lewis basic chalcogenide functional groups (i.e., S, Se, and Te), which show increased affinity toward heavy metals. Previous studies with metal chalcogenides revealed an improved affinity and selectivity toward heavy metals relative to their oxidic counterparts.^14–18^ However, the cation exchange process is slow in the bulk (grain size ≥ 1 μm) because the solid nonporous structure impedes the diffusion of ions, even if the reaction is thermodynamically favorable.^19^ 2-D (layered) materials such as layered double hydroxides intercalated with MoS_4_^–^ can undergo rapid sorption of Pb^2+^ between the sheets, demonstrating a removal capacity of 1.4 mmol Pb^2+^/g.^20–22^ An alternative approach used to great effect by Kanatzidis and co-workers is to exploit layered chalcogenide phases such as K_2*x*_Mn_*x*_Sn_3–*x*_S_6_ (KMS-1),^23^ K_2*x*_Mg_*x*_Sn_3–*x*_S_6_ (KMS-2),^24^ and K_*x*_[Bi_4–*x*_Mn_*x*_S_6_].^25^ Uptake of Pb^2+^ ions was achieved by exchange with metal cations (K^+^, Mn^2+^, and Mg^2+^) intercalated between the sheets, as well as ion sorption on the soft polysulfides that comprise the sheets, leading to removal capacities up to 1.65 mmol Pb^2+^/g.

Porous metal chalcogenide gels represent a promising 3-D architecture for heavy metal remediation. These include amorphous chalcogels pioneered by the Kanatzidis group and metal chalcogenide nanocrystal assemblies established by the Brock group.^26–28^ For example, porous amorphous Zn_2_Sn_*x*_S_2*x*+2_ (*x* = 1, 2) aerogels exhibited Pb^2+^ ion removal capacities of 1.53 mmol/g (*x* = 1) and 1.63 mmol/g (*x* = 2) operating via a mechanism of ion-exchange with Zn^2+^.^29^ However, the release of Zn^2+^ is problematic because it is toxic to microorganisms present in water bodies.^30^ Recently, the Islam group reported K–Co–Mo-S_*x*_ as a highly efficient amorphous chalcogel comprising Mo–S cluster units and Co–S polyhedra connected to form a negatively charged covalent Co–Mo–S network within which K^+^ ions are held by electrostatic forces.^31^ The K–Co–Mo-S_*x*_ chalcogel demonstrated 99.99% removal (≤5 ppb) of Pb^2+^ ion from a 100-ppb aqueous solution within 1 h, achieving a capacity of 5.53 mmol Pb^2+^/g (1146 mg Pb^2+^/g) based on a combination of ion exchange and sorption. As with the amorphous chalcogels, metal chalcogenide gels comprising interconnected nanocrystalline building blocks exhibit facile cation exchange reactions^32^ due to the much shorter diffusion path in nanocrystals vs bulk.^33^ The Brock group demonstrated the efficacy of ZnS nanocrystal assemblies linked by dichalcogenides (aerogels) for Pb^2+^ removal via cation exchange (principal mechanism) and ion chemisorption on the sulfur-rich walls of the pores. The combination of cation exchange and adsorption phenomena results in an impressive remediation capacity of 14 mmol Pb^2+^/g (2900 mg Pb^2+^/g). However, the reaction kinetics were slow for low Pb^2+^ concentrations; for a 100 ppb Pb^2+^ solution, more than 16 h were required to drive the concentration below 15 ppb (the EPA action limit). Moreover, as previously noted for the Zn_2_Sn_*x*_S_2*x*+2_ aerogels, liberated Zn^2+^ poses a concern for the environment.

In this work, a CaS nanoparticle aerogel was synthesized for the first time and evaluated for the remediation of heavy metals. Not only is Ca nontoxic compared to Zn, CaS has an exceptionally high *K*_sp_ value with the hardness of the Ca^2+^ ion thermodynamically favoring the cation exchange reaction with a soft Lewis acid.^34^ The synthesis of CaS gels is carried out by an oxidative assembly process where CaS nanoparticles are covalently linked by di or polysulfide bonds to form a porous framework that is then dried by supercritical CO_2_ extraction to obtain CaS aerogels.^35^ The CaS aerogels were tested for the removal of Pb^2+^ ions from water, exhibiting a fast rate of reaction for low concentrations, dropping from 100 to 5.4 ppb (below the EPA action limit of 15 ppb) within 1 h. In addition, the selectivity of CaS aerogels toward Pb^2+^ ions is high in the presence of 1 M Na^+^, Mg^2+^ as competing ions, or Ca^2+^ as an exchange inhibitor. Notably, the CaS aerogels exhibited a maximum removal efficiency of 17.1 mmol Pb^2+^/g aerogel (3543 mg/g), despite the fact that up to 50 wt % amorphous CaCO_3_ was discovered as a secondary phase. Moreover, by modifying the synthesis to reduce the amorphous CaCO_3_ content to ca. 15 wt %, the maximum capacity increased to 22.5 mmol Pb^2+^/g aerogel (4593 mg/g). In contrast, ambiently dried xerogels exhibit a maximum uptake of 40% Pb^2+^ (from 100 ppb solution) and saturate within 1 h. The fast removal rate, high efficiency, and selectivity of CaS aerogels toward Pb^2+^ ions make it a promising system for remediation.

## Materials and Methods

### Chemicals

Calcium acetate monohydrate (Ca­(CH_3_COO)_2_·H_2_O, 99%), tetramethylammonium hydroxide (TMAOH, 99%), 4-fluorothiophenol (FTP, 98%), oleic acid (90%), oleyl amine (98%), trioctylamine (98%), lead nitrate (Pb­(NO_3_)_2_), triethylamine (99%), tetranitromethane (TNM, 99%), and diphenyl thiourea (DPTU) (99%) were purchased from Sigma-Aldrich, USA. The microporous specimen capsules (30 μm pore size) were purchased from Electron Microscopy Sciences.

#### Safety Note

Tetranitromethane is an acute toxin and a suspected carcinogen that can lead to fire or explosion if it encounters combustible materials. Tetranitromethane should be stored in a refrigerator and diluted immediately before use. It should be handled in a well-ventilated hood free of combustibles, and the researcher should wear a flame-retardant lab-coat, safety goggles/glasses, and nitrile gloves and employ a safety shield.

### Synthesis of Oleate-Capped CaS Nanoparticles

Oleate-capped CaS nanoparticles were synthesized as described by Zhang et al. with some modifications.^36^ In a Schlenk flask, a mixture of 1 mmol (0.1769 g) of Ca­(CH_3_COO)_2_·H_2_O, oleyl amine (12 mL), oleic acid (2 mL), and trioctylamine (6 mL) was prepared, and the solution was purged with argon gas for 20 min. The mixture was heated to 120 °C for 30 min then 160 °C for 30 min in an argon flow to remove H_2_O and volatile substances and produce a transparent solution. The solution was allowed to cool down to room temperature, and a mixture of 10 mL of ethanol and 3.000 mmol (0.6849 g) of diphenyl thiourea (DPTU) was added to the flask by syringe. The ethanol was subsequently removed by heating the mixture to 90 °C and purging with a high flow of argon. The solution changed from colorless to yellow after 20 min. After the removal of ethanol, the mixture was heated to 320 °C for 1 h. After 20 min, the solution changed from a yellow color to white suspension. The reaction was stopped by removing heat and shutting off the gas flow. Then, the CaS nanoparticles were precipitated with ethanol and dispersed in 30 mL of cyclohexane. The purification process was repeated once more.

A modified synthesis was also performed in which the DPTU was increased to 3.168 mmol (0.7325 g), and the final heating step was performed at 330 °C. All other steps, including the purification, were unchanged.

### Ligand Exchange of CaS Nanoparticles with 4-Fluorothiophenolate (FTP)

#### In the Presence of Tetramethylammonium Hydroxide (TMAOH)

A mixture of 1.8 mL of 4-fluorothiophenol and 15 mL of ethanol was prepared. The pH of the solution was adjusted above 10 by the addition of TMAOH followed by ultrasonication for 20 min. The resultant thiolate solution was added to half of the oleate-capped nanoparticle solution described above (15 mL, ∼0.5 mmol Ca), and the mixture was ultrasonicated for 1 h in an ultrasonic bath. Ethyl acetate was then added to precipitate the FTP-capped CaS nanoparticles.

#### In the Presence of Triethylamine (TEA)

A solution of 1.8 mL of 4-fluorothiophenol and 3.2 mL of triethylamine (TEA) was prepared and ultrasonicated for 20 min. After ultrasonication, the pH of the mixture was 10.6. The mixture was then added to 20 mL of the oleate-capped nanoparticle solution described (∼0.67 mmol Ca), and the mixture was ultrasonicated for 1 h in an ultrasonic bath. The transparent sol became a white cloudy suspension instantly after addition of the oleate-capped CaS and was largely unchanged upon ultrasonication. The measured pH of the solution was 9.7. The FTP-capped CaS NPs were precipitated out and separated by centrifugation. The precipitate was dispersed in ethanol and precipitated with ethyl acetate. The washing was carried out once more, and the FTP-capped CaS NPs were dispersed in 30 mL of ethanol for the gelation process.

### Preparation of CaS Nanoparticle Wet Gels, Aerogels, and Xerogels^35^

A 0.3% TNM acetone solution was prepared by adding 3 μL of TNM to 1000 μL of acetone. 50 μL of this 0.3% TNM solution was added to half of the FTP-capped CaS sol prepared with TEA (15 mL, ∼0.33 mmol Ca), and the sol was left on the benchtop. After 1 h, a loose CaS gel was formed. Wet CaS gels were exchanged with acetone twice daily (performing five exchanges each time) for 5 days, placed in a 1.25 in. diameter sample holder with 100 μm stainless steel mesh on top and bottom, and dried in a Tousimis Autosamdri-931 Critical Point Dryer equipped with a 2.5 in. diameter chamber with 1.25 in. diameter chamber insert to prepare the CaS aerogel. The drying sequence is 1) 10 min slow fill of liquid CO_2_, 2) 10 min purge, 3) two 1-h stasis cycles, 4) heating to 36 °C for 10 min under supercritical conditions (>1072 psi), 5) slow pressure bleed to 550 psi, and 6) venting off. CaS xerogels were prepared for comparison to the aerogels by drying wet, solvent exchanged gels under ambient conditions (open vial, 2 days).

### Preparation of CaS Wet Gels and Aerogels from Oleate-Capped CaS NPs (without Ligand Exchange of Oleate for FTP)

A 20 mL portion of the oleate-capped CaS nanoparticle solution described (∼0.67 mmol Ca) dispersed in cyclohexane was taken in a 25 mL vial. 100 μL of 3% TNM solution was added to the CaS NP solution. After 20 h, the solution became cloudy and started forming a gel. The gel was then processed to form an aerogel, as described in the previous section.

### Qualitative Exchange of the CaS Wet Gel with Pb^**2**+^ Ion Solution

The cation exchange of the CaS wet gel with Pb^2+^ ion solution was carried out in a 1:3 molar ratio. The solution of Pb^2+^ ions was prepared by dissolving 1 mmol of Pb­(NO_3_)_2_ in 5 mL of nanopure water. Then, the Pb^2+^ solution was added slowly to the wet gel (∼0.33 mmol of Ca). Within 10 s, the white CaS gel converted to a fluffy loose black PbS gel. After 30 min, the supernatant was exchanged 5× with 5 mL of nanopure water to remove residual Pb­(NO_3_)_2_ from the solution. The PbS wet gel was then characterized by powder X-ray diffraction (PXRD) and transmission electron microscopy (TEM).

### Quantitative Exchange of CaS Aerogels and Xerogels with Pb^**2**+^ Ion Solution

#### 100 ppb Pb^**2**+^

A 1000 ppm Pb^2+^ solution was prepared by dissolving 0.032 g of Pb­(NO_3_)_2_ in nano pure water to a volume of 20 mL. This solution was then diluted by a factor of 10^4^ to produce the 100 ppb Pb^2+^ solution. Aiming for a ratio of ∼1000 mL of Pb^2+^ solution/1000 mg CaS aerogels, an average (from three independent samples) of 10.55 ± 0.14 g (∼10 mL) of Pb^2+^ solution was added to 10.15 ± 0.32 mg of CaS aerogels (also averaged over three samples). The individually weighed CaS aerogel samples were placed in a microporous capsule (30 μm pore size) that was subsequently placed in a vial containing the 100 ppb solution of Pb^2+^. At time intervals of 0.1, 0.2, 0.3, 0.4, 0.5, 1, 4, 6, 24, and 36 h, 100 μL of the supernatant solution was removed using a syringe equipped with a 100 μm syringe filter and weighed in a tared falcon tube, which was then diluted with a mixture of 2% nitric and 0.5% hydrochloric acid solution to a well-defined mass (∼10 g) using a microbalance (precision to 0.0001 g). The solutions were then analyzed by ICP-MS, collecting three runs (Supporting Information Calculation S1, S1.1, and S1.2), and the average values were plotted to determine the removal rate.

CaS xerogels were also evaluated. An average of 10.46 ± 0.17 g (≈10 mL) of Pb^2+^ solution was added to 10.34 ± 0.12 mg of CaS xerogels (values averaged from two independent samples, Supporting Information Calculations S1.3.1 and S1.3.2.)

#### 20, 200, 2000, and 20,000 ppm Pb^**2**+^

A 20,000 ppm solution was prepared by dissolving 1.59 g of Pb (NO_3_)_2_ in nano pure water to a volume of 50 mL. 10, 100, and 1000 μL of the 20,000 ppm Pb^2+^ solution were diluted with nano pure water to an appropriate mass to make 20, 200, and 2000 ppm Pb^2+^ solutions, respectively. Aiming for a ratio of ∼1000 mL of Pb^2+^ solution/1000 mg CaS aerogels, 10 ± 0.5 mL of Pb^2+^ solution was exchanged with 10.4 ± 0.87 mg of CaS aerogels (standard deviation represents the average of two independent samples). The weighed CaS aerogel samples were placed in four microporous capsules and then transferred to four vials containing 10 mL of 20, 200, 2000, and 20,000 ppm Pb^2+^ solutions, respectively. Aliquots were collected after 1, 3, 6, 24, and 36 h time intervals as described for the 100 ppb sample. 100 μL aliquots were separated and analyzed by ICP-MS as described above for the 100 ppb solution (Supporting Information Calculations S2–S5).

### Study of Pb^**2**+^ Removal in the Presence of Competitive Ions

The removal of Pb^2+^ was studied in the presence of competing ions (i.e., Na^+^, Ca^2+^, and Mg^2+^) with a *V*/*m* ratio of 1000 mL/g using the batch method at room temperature and a contact time of 24 h. The competing ions were present in 1000-fold excess (1 M competing ion vs 1 mM Pb^2+^), and the experiments were carried out for low and high Pb^2+^ concentrations.

The distribution coefficient (*K*_d_) measures the affinity and selectivity of CaS aerogels for Pb^2+^ removal quantitively using the following equation:

$$K_{d}=\frac{V}{m}\left(\frac{C_{0}-C_{f}}{C_{f}}\right)$$

where *C*_0_ and *C*_f_ represent the initial and equilibrium (final) metal ion concentrations, respectively, expressed in ppm (mg L^–1^) or ppb (μg L^–1^). *V* is the volume of the test solution (mL), and *m* is the mass of the solid adsorbent (g) used in the experiment.

The adsorption capacity (*q*_e_) of the CaS aerogel for Pb^2+^ removal was calculated using

$$q_{e}=\frac{V}{m}(C_{0}-C_{f})$$

where the variables are defined as above, with *C*_0_ and *C*_f_ expressed in ppm (mg L^–1^), and *V* expressed in liters (L).

### Characterization

#### Powder X-ray Diffraction (PXRD)

The diffraction patterns of CaS nanoparticles and CaS wet gels and aerogels before and after cation exchange with Pb^2+^ ions were measured using a Bruker D2 phaser with Cu K_α_ (λ = 1.514 Å) radiation (anode source operating at 40 mV and 150 mA). The sample was prepared by drop casting a suspension of CaS nanoparticles, gels, or exchanged gels in methanol on a zero-background quartz holder. The diffraction peaks were indexed relative to the Powder Diffraction File database (**PDF-01–071–4760**).

#### Rietveld Refinement

Rietveld refinement of powder X-ray diffraction data collected on a CaS aerogel annealed at 800 °C in argon flow (10 °C/min ramp rate) was performed using GSAS-II (Supporting Information). The histogram scale factor was refined to align the calculated intensities with the experimental pattern. Background coefficients were adjusted to 10 to remove nonstructural contributions. Sample displacement was corrected to account for minor misalignment during the measurement. Peak broadening was modeled by using the Caglioti equation through refinement of the U, V, and W parameters. Unit cell parameters were refined to match the calculated peak positions with observed data. Atomic coordinates and related structural parameters were optimized at the phase level. Phase fractions were refined to determine the proportion of each crystalline phase.

#### High-Resolution Transmission Electron Microscopy (HR-TEM) and High-Angle Annular Dark-field Scanning Transmission Electron Microscopy (HAADF-STEM) with In Situ Energy-Dispersive X-ray Spectroscopy (EDS)

The nanoparticles and aerogels were imaged by HR-TEM, HAADF-STEM; chemical mapping and atomic composition were obtained by EDS. Data were acquired on a Thermo Fisher Talos F200X G2 Scanning Transmission Electron Microscope with an acceleration voltage of 200 kV. Samples were prepared by ultrasonicating in acetone and ethanol followed by drop casting on a carbon film supported on a copper grid (200 mesh, 3.05 mm) bought from SPI Supplies. The grids were placed in a grid box to protect them from contamination and stored in a vacuum desiccator to minimize oxidation.

#### Surface Area and Pore Size Analysis

The surface areas and pore characteristics of CaS aerogels and a xerogel were determined by nitrogen physisorption using a Micromeritics TriStar II instrument, and the data were processed by Micromeritics TriStar II 3020 v.3.02 software. ∼10 mg of CaS aerogels or the xerogel was degassed over 14 h under N_2_ gas flow at 120 °C before physisorption measurements. The surface areas of the CaS aerogels and the xerogel were calculated by the Brunauer–Emmett–Teller (BET) method, and the pore size distribution was determined using the Barret–Joyner–Halenda (BJH) method.

#### Inductively Coupled Plasma Mass Spectroscopy (ICP-MS)

The concentration of Ca^2+^ and Pb^2+^ in solution during cation exchange was measured by an Agilent 7700 ICP-MS instrument. The data were collected as counts per second. Standard calibration curves for Ca and Pb were constructed by preparing dilute solutions of 1, 5, 20, 30, 50, 100, 150, and 200 ppb from 10 ppm standard samples bought from Inorganic Ventures. To analyze instrument drift, the standards were measured before and after the samples. Blank samples were analyzed periodically between samples to check for sampling probe contamination.

#### Fourier Transform Infrared Spectroscopy (FTIR)

Fourier transform infrared spectroscopy (FTIR) was used to analyze the functional groups of ligands present on the surface of CaS nanoparticles before and after ligand exchange with FTP, as well as residual ligands on the CaS gel after oxidative assembly. A spectrum of 4-fluorothiophenol was collected for reference. A spectrophotometer (Bruker Tensor 27) with an ATR (Invenio R) and Opus 8.7 software was used to obtain the FTIR spectra of the dried solid CaS NPs and aerogels.

#### Nuclear Magnetic Resonance Spectroscopy (NMR)

^1^H NMR data of CaS nanoparticles capped with oleate or FTP were collected on a Bruker Avance NEO 500 MHz spectrometer using Bruker TopSpin 4.1 software. Samples were prepared by dispersing 2 mg of oleate-capped CaS NPs in 2 mL of D-toluene or 2 mg of FTP-capped CaS NPs in 2 mL of D-ethanol; the solutions were transferred to NMR tubes for measurement.

#### Thermogravimetric Analysis

Thermogravimetric analysis (TGA) was carried out using a TA Instruments Q50 instrument with weight precision ±0.01%, sensitivity 0.1 μg, and isothermal temperature accuracy ±1 °C. The instrument was kept in a glovebox to maintain an inert atmosphere. The CaS aerogel sample was heated from 27 to 1000 °C with a ramp rate of 10 °C/min in a nitrogen atmosphere.

#### X-ray Photoelectron Spectroscopy (XPS)

The X-ray photoelectron spectroscopy (XPS) data were recorded from a Fisher Scientific Nexsa, model no: NXA9952425 with an instrument pass energy of 50 eV. The photoelectron source was Al Kα with a flood gun of 100 eV. The analysis spot size was 400 μm. The high-resolution data for O 1s, S 2p, Ca 2p, C 1s, N 1s, and F 1s were recorded from the instrument and deconvoluted with Thermo Fisher Avantage software.

## Results

### Synthesis and Characterization of CaS Gels, Aerogels, and Xerogels

CaS gels were prepared from the oxidative assembly of 4-fluorothiophenolate-capped CaS nanocrystals according to Scheme 1. Oleate-oleyl amine-capped CaS nanoparticles (NPs) were prepared by modification of a published procedure in which calcium acetate is dissolved in oleyl amine, oleic acid, and trioctylamine followed by introduction of the sulfur source (diphenylthiourea) and heating to 320 °C for 1 h.^36^ The particles are cube-shaped with an average size of 20 ± 2.9 nm (dimension of cube body diagonal), as determined by TEM (Figure S1), and the particles adopt the CaS (Zincblende) structure based on powder X-ray diffraction (PXRD), with no evidence of secondary crystalline phases (Figure 1). The crystallite size was calculated to be 16.2 ± 0.5 nm by the application of the Scherrer equation to the data in Figure 1.

**Scheme 1:**
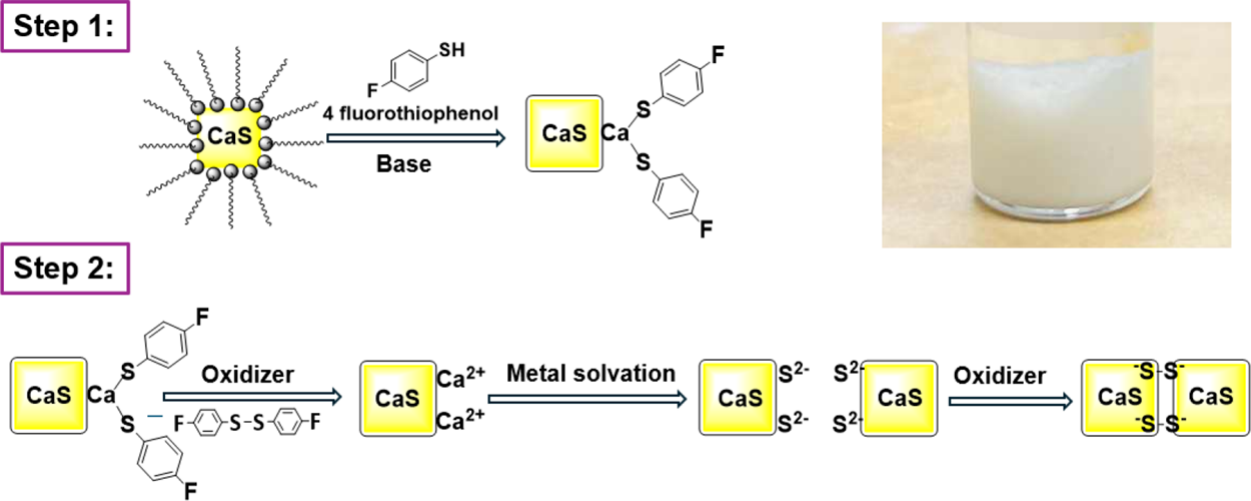
Synthesis of CaS Nanoparticle Gels^a^

a Step 1: Ligand exchange of oleate-capped CaS nanoparticles with 4-fluorothiophenolate (FTP). Step 2: Oxidative assembly of FTP-capped CaS NPs by oxidative removal of the ligand as disulfide, Ca^2+^ solvation, and oxidative crosslinking to produce disulfide-linked gels. The photo shows an illustration of a CaS wet gel in a 1 cm diameter vial.

**Figure 1:**
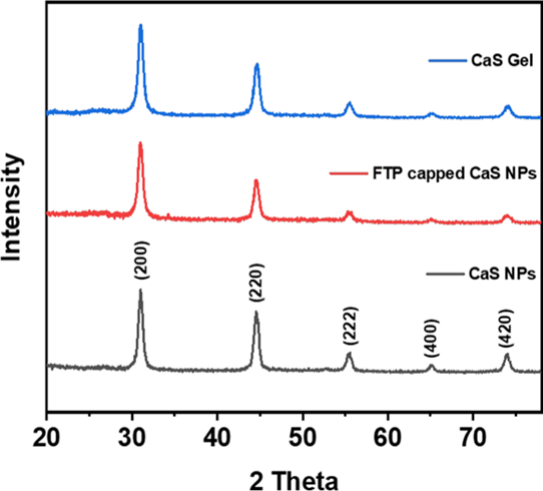
PXRD of CaS NPs, CaS NPs capped with 4-fluorothiophenolate (FTP) and CaS gels.

In the first step, the oleate ligands (possibly accompanied by oleyl amine ligands from the original synthesis) are exchanged with 4-fluorothiophenol in the presence of a base to deprotonate the thiol. Initially, TMAOH was used as the base; however, the resultant FTP-capped CaS NPs were accompanied by crystalline CaCO_3_ formation as evidenced by PXRD (Figure S2) and corroborated by STEM and EDS (Figure S3 and Table S1). These data suggest the hydroxide displaces sulfide to form Ca­(OH)_2_ species, which subsequently undergo insertion of atmospheric CO_2_ to form the carbonate.^39^ Accordingly, an amine base (triethylamine) was employed instead, delivering FTP-capped CaS nanoparticles with no evident crystalline carbonate impurity (Figure 1). The successful removal of oleate and installation of FTP were confirmed by ^1^HNMR (loss of oleate vinylic peak (Figure S4)) and supported by FTIR (Figure S5) (please see detailed interpretation in the Supporting Information). The FTIR also shows a small peak at 873 cm^–1^ that is attributed to amorphous CaCO_3_ formation. This peak is also present in the oleate-capped CaS nanocubes (Figure S5), suggesting that amorphous CaCO_3_ is a byproduct of CaS nanocube synthesis. XPS data acquired on oleate-capped CaS nanocubes (Figure S6) are consistent with this interpretation (see associated discussion in the Supporting Information).

In the second step (Scheme 1), the FTP-capped CaS NPs are oxidized with tetranitromethane to remove the FTP (deprotect the surface) as disulfide and expose undercoordinated Ca^2+^ ions. Solvation of Ca^2+^ reveals surface sulfide, which is oxidized to form interparticle disulfide bonds, producing a highly porous 3D nanoparticle network of CaS (gel).^35^ The PXRD data after ligand exchange with FTP, and after oxidative assembly of FTP-capped CaS NPs, are essentially identical to those of the original oleate-capped CaS NPs (Figure 1); however, FTIR data (Figure S5) suggest that amorphous CaCO_3_ present in oleate- and FTP-capped CaS nanoparticles also persists in the gel.

The morphology of the CaS wet gel is shown in the TEM micrograph of Figure 2a, and HAADF-STEM imaging along with EDS composition mapping is shown in Figure 2b–e. The cube-shaped CaS NPs form a 3-D porous network, where individual CaS NPs can be seen to connect via different orientations. The EDS mapping of Ca and S shows that Ca and S are colocalized over the cube-shaped features in the HAADF-STEM image, with atomic percentages of 55.34 ± 5.44% (Ca) and 45.15 ± 5.44% (S) corresponding to a Ca:S ratio of 1.23 (Table S2). Additionally, a small amount of F (0.51 ± 0.33%) is also detected, attributed to residual FTP ligands. Elemental analysis and line-scan profiling of the CaS gel (Figure S7) also show a Ca-rich composition relative to that of sulfur, and this is accompanied by a persistent oxygen signal spatially correlated with calcium. The concurrent presence of Ca and O across nanoparticle-rich regions and interparticle interfaces is indicative of the partial formation of CaCO_3_ on CaS nanoparticle surfaces, consistent with the FTIR data (Figure S5).

**Figure 2:**
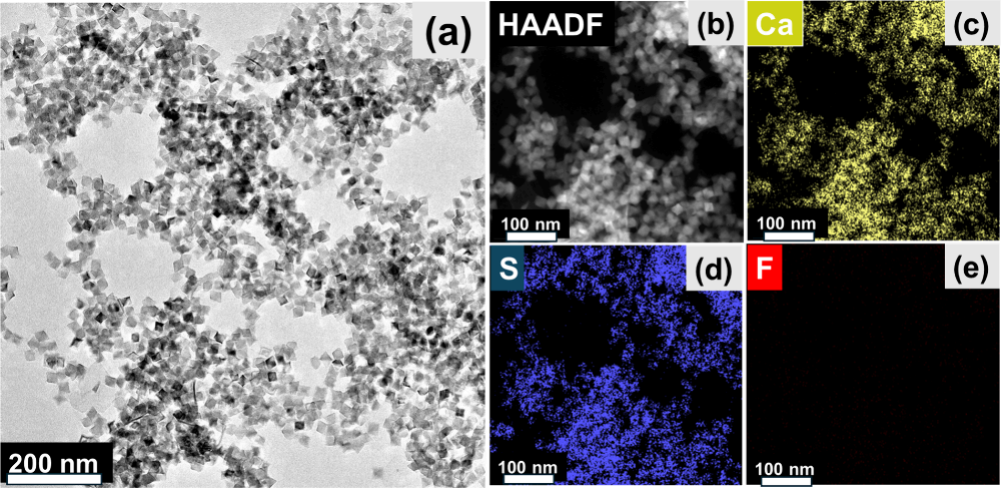
(a) TEM image of a CaS gel; (b) HAADF-STEM image of a CaS gel; and EDS mapping of (c) Ca, (d) S, and (e) F.

CaS aerogel formation is achieved by supercritical CO_2_ drying of the CaS wet gels, in which wet gels are first exchanged with liquid CO_2_, followed by an increase in temperature to drive the supercritical transition and then venting. The supercritical CO_2_ eliminates capillary forces that give rise to pore collapse upon solvent evaporation. For comparison, ambient-pressure drying was also conducted, producing CaS xerogels.

To quantify the amount of residual ligands and carbonate in the aerogels, thermogravimetric analysis was performed on the CaS aerogel under an inert atmosphere (Figure 3). Losses occurring at temperatures below 460 °C (Stages 1–3, 12.6 wt %) are attributed to loss of residual ligands (and possibly some adsorbed water in Stage 1), whereas the loss between 460 and 710 °C (Stage 4, 19.5%) is attributed to decomposition of CaCO_3_ to produce CaO and eliminate CO_2_. To confirm the attribution of Stage 4 to CaCO_3_ decomposition, a fresh sample of CaS aerogel was heated to 800 °C in a tube furnace under argon flow and then characterized by PXRD, revealing a combination of CaS and CaO phases. Rietveld refinement of the data (Figure 4) yielded calculated percentages of 70.1% CaS and 29.9% CaO. These data are close to the values computed from TGA weight loss (Calculation S6): 63.3% CaS and 36.7% CaO, consistent with our interpretation. Thus, the CaS aerogels actually comprise ∼50% CaCO_3_ impurity, carried through from the original CaS nanocube synthesis.

**Figure 3:**
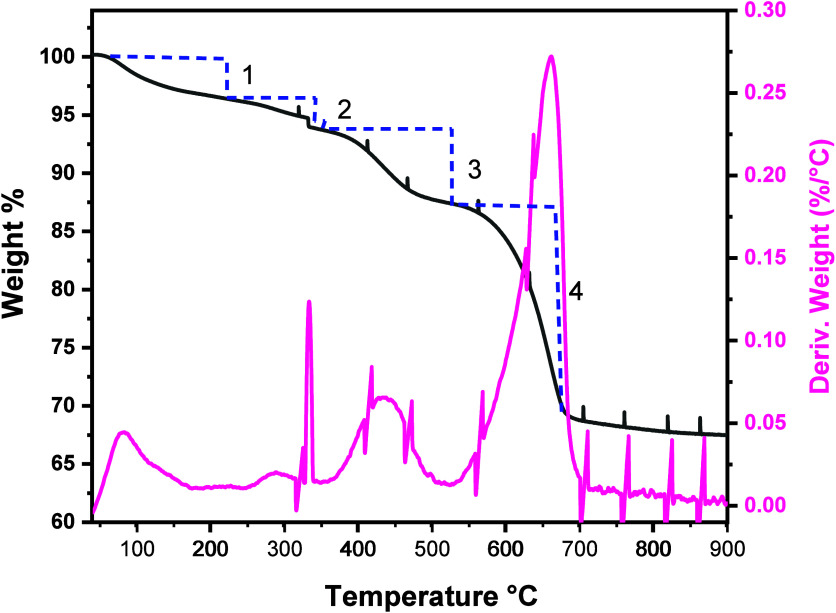
Thermogravimetric analysis of a CaS aerogel where the weight loss with temperature and the derivative weight with temperature are shown in black and pink, respectively, and the stages of weight loss are numbered 1, 2, 3, and 4. The weight losses in stages 1 (47–200 °C), 2 (200–326 °C), 3 (326–460 °C)**,** and 4 (460–710 °C) are 3.2, 2.8, 6.6, and 19.5%, respectively.

**Figure 4:**
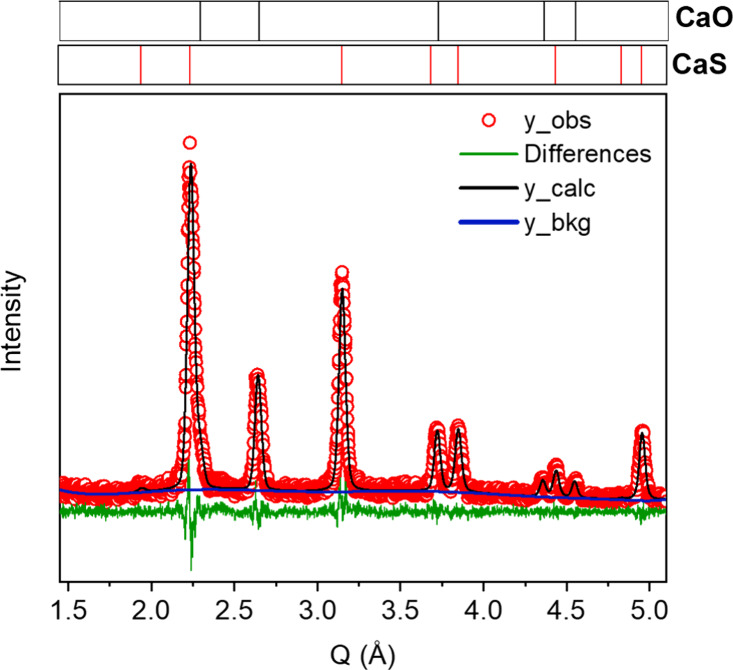
Rietveld refinement of PXRD data acquired on a CaS aerogel after heating at 800 °C under an argon flow. See the Supporting Information for refinement data.

XPS data was acquired to elucidate the surface composition and bonding environments within the CaS gel produced from FTP-capped CaS nanoparticles (Figure 5). The C 1s spectrum exhibits multiple components corresponding to C–C/C–H (∼284.8 eV), C–O (∼286.2 eV), and carbonate-related species (∼289–290 eV), consistent with the presence of organic ligands and partial surface carbonation. However, no F is detected in the XPS, indicative of minimal residual FTP (consistent with FTIR, Figure S5, and EDS, Table S2 and Figure S7), nor is there any N signal, suggesting no amine is carried over from the ligand exchange. The Ca 2p region displays well-resolved spin–orbit doublets attributable to Ca–S and Ca–CO_3_ environments. The coexistence of these environments is consistent with a gel framework comprising mixed CaS/CaCO_3_ phases rather than a single homogeneous sulfide.

**Figure 5:**
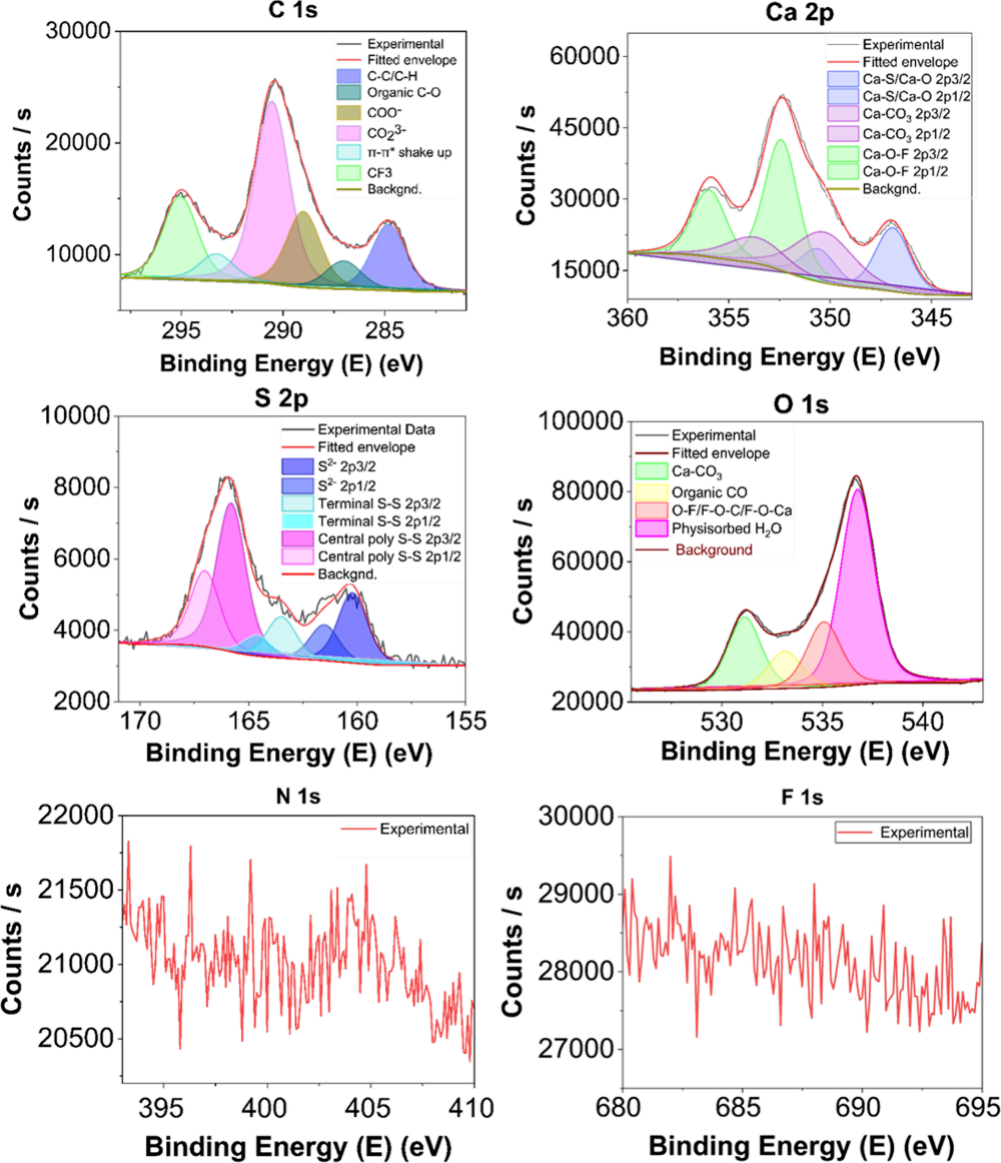
X-ray photoelectron spectroscopy (XPS) analysis of the 4-fluorothiophenolate (FTP)-capped CaS gel. High-resolution spectra are shown for the C 1s, Ca 2p, S 2p, O 1s, F 1s, and N 1s regions. Solid lines represent experimental data, and colored components correspond to fitted chemical states.

Most notably, the S 2p spectrum provides direct insight into the gelation mechanism. In addition to sulfide (S^2–^) doublets characteristic of CaS, distinct higher-binding-energy components are observed and assigned to terminal and central polysulfide (S–S) species (163.5 and 164.7 eV).^37^ The presence of these S–S bonding motifs is consistent with the oxidative assembly of surface sulfide during gelation and supports the proposed interparticle coupling mechanism.

Nitrogen adsorption–desorption isotherms and pore size distributions for CaS aerogels (∼50 wt % CaCO_3_) are shown in Figure 6. The isotherm is Type IV with a pronounced hysteresis loop (Figure 6a), indicating a mesoporous structure formed by interparticle voids within the gel networks. The BET surface area is 143.6 m^2^/g, and the pore size distributions derived from the adsorption branch reveal pores spanning a mesoporous range of approximately 2–30 nm (Figure 6b). In contrast, ambiently dried CaS xerogels exhibit a lower surface area of 106.4 m^2^/g and the pore size distribution is much narrower, extending from 3 to 13 nm (Figure S8). The absence of a broad tail toward larger pore diameters implies a compact and relatively narrow pore size distribution, consistent with structural shrinkage during ambient drying. Overall, these data suggest that the oxidative assembly of CaS is a robust process, even in the presence of significant carbonate impurities that might be expected to inhibit gelation.

**Figure 6:**
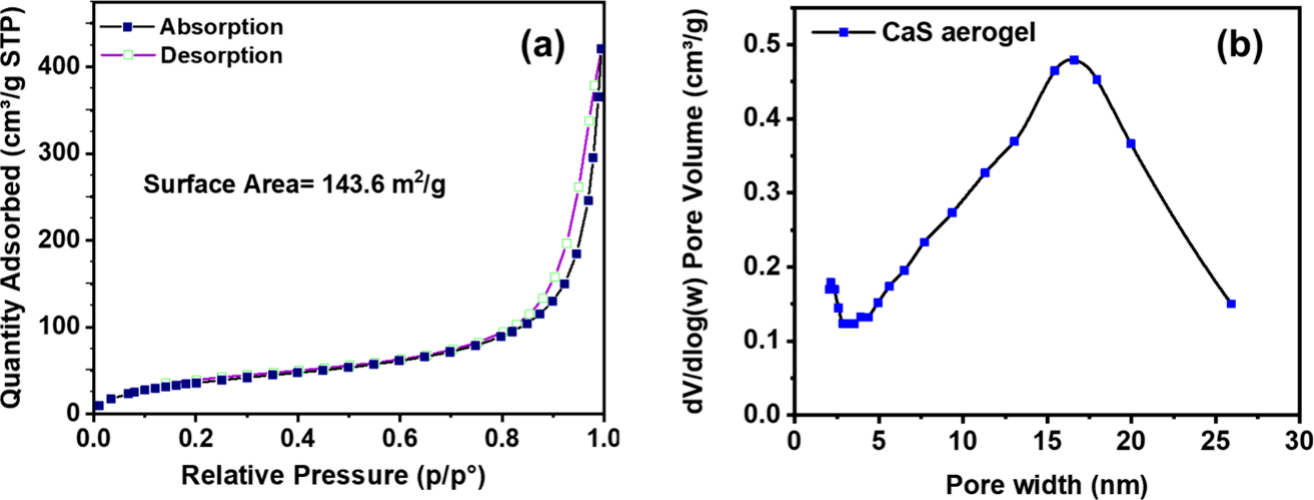
(a) Nitrogen adsorption and desorption isotherms of the CaS aerogel. (b) Pore size (based on the BJH model) distribution within the CaS aerogel.

### Reducing Carbonate Impurity Formation

The synthesis process for CaS nanocubes was modified by increasing the DPTU (sulfur precursor) to 3.168 mmol (0.7325 g) and increasing the temperature to 330 °C. The resultant CaS aerogels were found to have ∼15% CaCO_3_ from the TGA data (Figure S9 and Calculation S7). HAADF-STEM (Figure S10a) imaging shows a highly interconnected three-dimensional network composed of nanoscale primary particles, and STEM-EDS elemental mapping (Figure S10b–f) reveals homogeneous spatial distributions of Ca and S throughout the gel framework, with a Ca:S ratio of 1.09, significantly decreased from the value of 1.23 obtained for the CaS aerogels with ∼50 wt % CaCO_3_. The porous architecture is also reflected in the results from gas sorption analysis (Figure S8) where a BET surface area of 161.1 m^2^/g is obtained and the pore-size distribution spans a mesoporous range of approximately 2–30 nm, with a higher cumulative pore volume compared to CaS gels containing ∼50% CaCO_3_ content. Together, these results demonstrate that reducing the CaCO_3_ content to ∼15% preserves strong network connectivity and enhances accessible porosity within the CaS gel.

### Qualitative Cation Exchange of Pb^2+^ with the CaS Wet Gel (∼50 wt % CaCO_3_)

A qualitative cation exchange reaction was conducted with the CaS wet gel by taking a 1:3 molar ratio of the CaS wet gel (based on the original amount of calcium acetate used in the synthesis) to Pb­(NO_3_)_2_ solution in nanopure water. The exchange was very fast, and within 10 s, the gel started changing color from white (CaS) to black (PbS) and became completely black after 30 s. After 30 min, the sample was washed 5 times with nanopure water and analyzed by PXRD and STEM-EDS mapping to evaluate the phase and composition, respectively. Figure 7 shows the PXRD of CaS NPs, the CaS wet gel before exchange, and the PbS wet gel after the CaS exchange with Pb^2+^ ions. The PXRD pattern collected after Pb^2+^ treatment matches the pattern for bulk PbS, consistent with the full exchange of the nanocrystalline CaS gel to make the nanocrystalline PbS gel. The TEM and HAADF-STEM imaging of the gel after Pb^2+^ exchange shows the particles remain connected (Figure 8a,b), suggesting that the 3-D gel network remains intact upon exchange. STEM EDS mapping shows the Pb and S signals to be colocalized, with no detectable Ca (Figure 8c–e), consistent with full exchange of CaS. HAADF-STEM images (Figure S11) reveal low contrast areas, indicative of an amorphous phase. These regions have measurable Pb, but very little S (Pb:S = 14.4) and a significant O signal (Pb:O = 4.2). These data are consistent with amorphous PbCO_3_ formation upon the exchange of amorphous CaCO_3_ with Pb. Thus, both the calcium sulfide and carbonate components undergo exchange with Pb. Based on this qualitative study, we set out to conduct a quantitative study to probe the efficiency for remediation of Pb^2+^ by conducting a series of reactions with different concentrations of Pb^2+^ ions.

**Figure 7:**
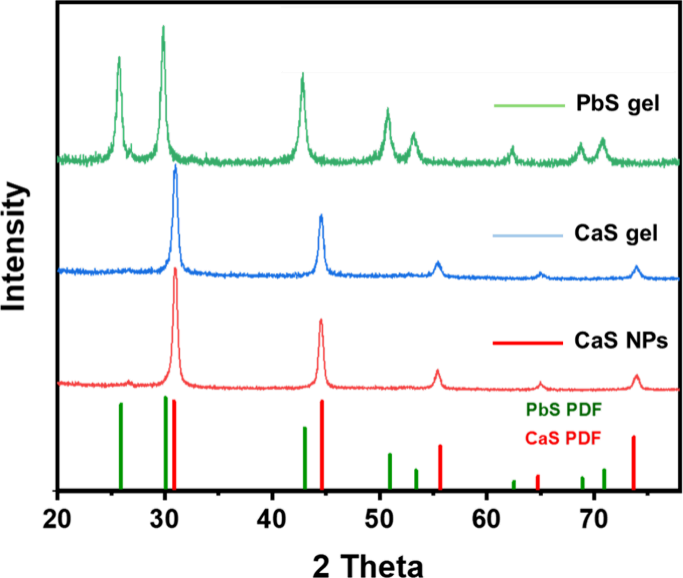
PXRD of CaS NPs, the CaS wet gel (∼50 wt % CaCO_3_), and the CaS wet gel after exchange with Pb^2+^ to produce a PbS wet gel (PbS reference: PDF-01–078–1901).

**Figure 8:**
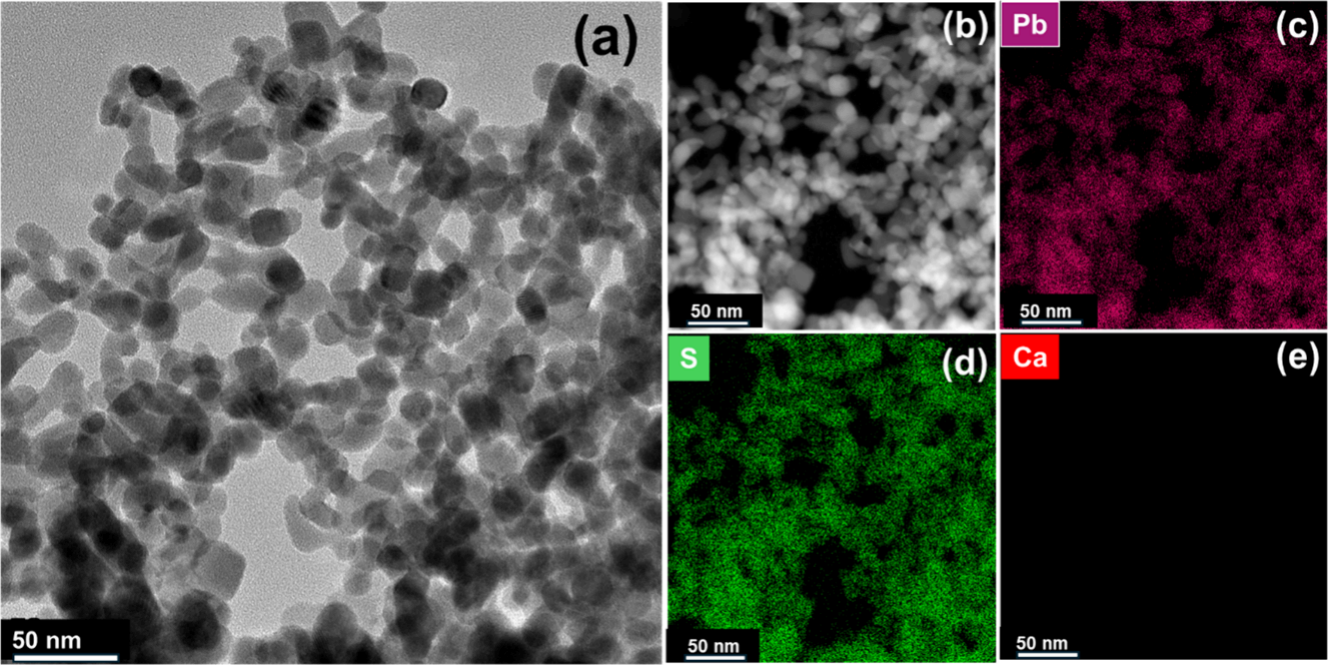
(a) TEM image of a PbS gel produced by exchange of a CaS gel with Pb^2+^; (b) HAADF-STEM image of the PbS gel; and EDS mapping of (c) Pb, (d) S, and (e) Ca.

### Quantitative Exchange of the CaS Aerogel (∼50 wt % CaCO_3_) with Pb^**2**+^ (100 ppb)

The quantitative study of the exchange of the CaS aerogel was carried out with an aqueous solution of Pb^2+^ ions with concentration of 100.56 ± 4.50 parts per billion (ppb). A microporous capsule with 30 μm pores was used to contain the CaS aerogel. The capsule with the CaS aerogel was added to the aqueous Pb^2+^ ion solution. At specific time intervals, aliquots were taken for ICP-MS to determine the concentrations of the remaining Pb^2+^ ions and the Ca^2+^ ions released. Three individual experiments were conducted (Calculation S1). The mean values of Ca^2+^ (Calculation S1.1) and Pb^2+^ (Calculation S1.2) concentrations from the three runs as a function of time (up to 36 h) are plotted in Figure 9a. Within 1 h, the concentration of Pb^2+^ in solution was 5.38 ± 0.68 ppb, which is below the action limit (15 ppb in drinking water). The concentration of Pb^2+^ significantly decreased over 36 h to become 1.91 ± 0.56 ppb, corresponding to removal of 98% of Pb^2+^ ions. The concentration of Ca^2+^ also increased significantly with time, which is consistent with ion exchange. The above results depict a promising heavy metal removal efficiency of the synthesized CaS aerogel for low concentrations of the Pb^2+^ ion. In contrast, the corresponding xerogel (ambiently dried gel) became saturated within the first few hours, only removing ca. 40% of the Pb^2+^ ions (Figure 9b).

**Figure 9:**
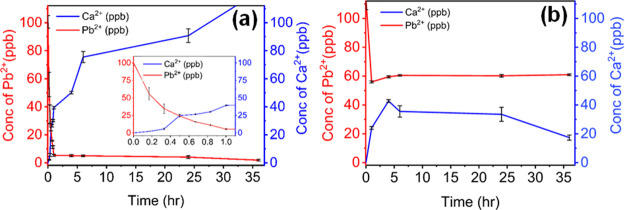
Treatment of water containing low concentration Pb^2+^ (100 ppb) with (a) CaS aerogel (∼50 wt % CaCO_3_) and (b) xerogel. The inset in (a) shows an expanded view of the first hour of exchange. The error bars represent the standard deviations from 3 runs for the aerogel and 2 runs for the xerogel.

Table 1 shows the removal capacity and distribution coefficient (*K*_d_) of a CaS aerogel (50 wt % CaCO_3_) for a 100 ppb Pb^2+^ solution after 36 h of exchange. The removal percentage and *K*_d_ value of Pb^2+^ removal are 98.1% and 4.9 × 10^4^ mL/g, respectively. These data indicate that the CaS aerogel Pb^2+^ remediation performance is “very good” (>5 × 10 ^3^) to “outstanding” (>5 × 10^4^).^33^

**Table 1:** Quantity (mmol), Percentage, and Distribution Coefficient, *K*_d_ (mL/g), for the Removal of 100 ppb Pb^2+^ Ion by 10 mg of CaS Aerogel After 36 h

**initial Pb** ^**2+**^ **concentration (ppb)**	**final Pb** ^**2+**^ **concentration (ppb)**	**amount of Pb** ^**2+**^ **removed** (mmol/mL)	**removal (%)**	***K***_**d**_ **(mL/g)**
100.56 ± 4.50	1.91 ± 0.56	0.0000048	98.1	4.9 × 10^4^

### Quantitative Exchange of the CaS Aerogel (∼50 wt % CaCO_3_) with Pb^**2**+^ at Higher Concentrations (20, 200, 2000 ppm)

The calculations from 2 individual experiments conducted with 20, 200, and 2000 ppm of Pb^2+^ solution and CaS aerogel are shown in the Supporting Information (Calculations S2–S4). The mean value is the standard deviation of Ca^2+^ (Calculations S2.1, S3.1, and S4.1), and Pb^2+^ (Calculations S2.2, S3.2, and S4.2) concentrations vs time intervals are plotted in Figure 10. As with the low concentration case (100 ppb), the concentration of Pb^2+^ ions in 20, 200, and 2000 ppm decreases over the 36 h run and the concentration of Ca^2+^ ions increases over the same period. Removal efficiencies remain above 95%, and *K*_d_ values are comparable to the case for 100 ppb ((2–5) × 10^4^, see Table 2).

**Figure 10:**
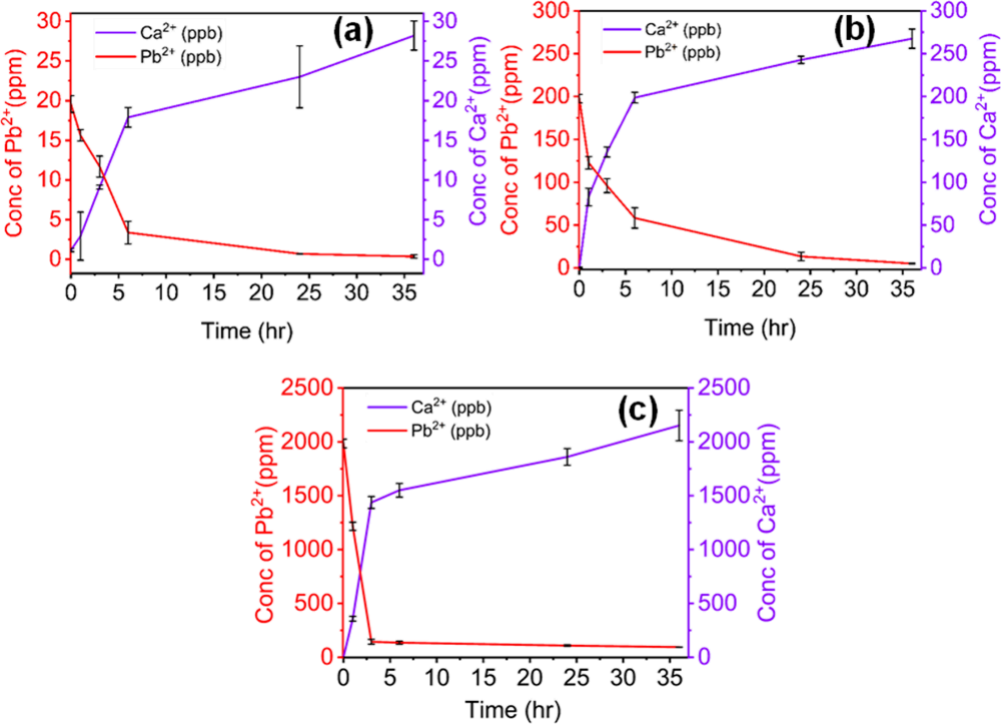
Treatment of water containing Pb^2+^ with CaS aerogels comprising ∼50 wt % CaCO_3_. (a) 20 ppm, (b) 200 ppm, and (c) 2000 ppm Pb^2+^. The error bars represent the standard deviations from 2 runs.

**Table 2:** Quantity of Pb^2+^ and Ca^2+^ (ppm and mmol), Removal Percentage of Pb^2+^, and the Distribution Coefficient, *K*_d_ (mL/g), for the Removal of 20–2000 ppm Pb^2+^ Ions by 10 mg of CaS Aerogel (∼50 wt % CaCO_3_)

**initial Pb** ^**2+**^ **concentration (ppm)**	**final concentration after 24 h (ppm)**	**amount of Pb** ^**2+**^ **removed** (mmol/mL)	**initial Ca** ^**2+**^ **concentration (ppm)**	**final concentration after 24 h (ppm)**	**amount of Ca** ^**2+**^ **ion liberated**(mmol/mL)	***K***_**d**_ (mL/g) values of Pb^2+^ ion removal	**Removal (%)**
19.57 ± 1.05	0.36 ± 0.21	9.3 × 10^–4^	1.16 ± 0.18	29.49 ± 2.51	13.1 × 10^–4^	5.43 × 10^4^	98.16
197.64 ± 4.90	5.09 ± 0.49	9.3 × 10^–3^	0.71 ± 0.45	267.52 ± 11.21	12.8 × 10^–3^	3.83 × 10^4^	97.42
1984.04 ± 1.24	94.27 ± 1.41	9.1 × 10^–2^	0.37 ± 0.11	2151.19 ± 141.37	10.03 × 10^–2^	2.03 × 10^4^	95.24

To find out the highest capacity of Pb^2+^ ion removal, exchange of CaS with 20000 ppm Pb^2+^ ion solution was evaluated in two separate runs (See Calculation S5) and the mean and standard deviations of Ca^2+^ (Calculation S5.1) and Pb^2+^ (Calculation S5.2) were monitored as a function of time. Note that for these runs, the moles of Pb^2+^ in the 10 mL of solution are 7-fold greater than Ca^2+^ found in the 10 mg of the CaS aerogel used in the exchange. From Table 3, the removal capacity of Pb^2+^ ions is 17.1 mmol of Pb^2+^/g of the CaS aerogel (3538 mg/g). This is far greater than the theoretical value of 2464 mg of Pb/g of CaS (calculated considering 50 wt % CaCO_3_) for complete Ca^2+^ exchange (Calculation S8).

**Table 3:** Quantity of Pb^2+^ and Ca^2+^ (ppm and mmol), Removal Capacity of Pb^2+^ (mg Pb/g CaS and mmol Pb/g CaS) for the Removal of 20,000 ppm Pb^2+^ Ions by 10 mg of CaS Aerogel (∼50 wt % CaCO_3_)

**initial Pb** ^**2+**^ **concentration (ppm)**	**final Pb** ^**2+**^ **concentration (ppm)**	**amount of Pb** ^**2+**^ **removed (mmol)**	**amount of Ca** ^**2+**^ **in CaS (mmol)**	**removal capacity (mg Pb/g CaS)**	**removal capacity (mmol Pb/g CaS)**
19999.16 ± 171.16	16511.15 ± 260.38	0.168 ± 0.015	0.149 ± 0.016	3538 ± 11.26	17.07 ± 0.01

### Effect of CaCO_3_ Content on Pb^**2**+^ Removal by CaS Aerogels

Reducing the CaCO_3_ content in the CaS gel from ∼50 to ∼15% leads to a pronounced enhancement in the Pb^2+^ removal capacity. The CaS gel with ∼15% CaCO_3_ removes Pb^2+^ from an initial concentration of 20,778 ± 128 to 16,114 ± 154 ppm, achieving a substantially higher removal capacity of 4664 ± 21 mg g^–1^ (22.50 ± 0.02 mmol g^–1^). Although the BET surface area (Figures 6 and S8) increases only modestly between the two samples (from 143.6 to 161.1 m^2^/g), the CaS gel with lower wt % CaCO_3_ exhibits a significantly higher total pore volume (Figure S8), which enhances Pb^2+^ transport into the interior of the gel. This suggests that Pb^2+^ uptake is governed primarily by pore accessibility and network openness rather than surface area alone, explaining the superior performance of the CaS aerogel containing ∼15 wt % CaCO_3_.

### Effect of Competing Ions on Removal Efficiency

The presence of competing ions such as Na^+^ and Mg^2+^ in high concentrations (1 M), which are expected to be present in natural sources of water, does not show significant changes in the Pb^2+^ removal efficiency of CaS aerogels (50 wt % CaCO_3_), nor do high concentrations of Ca^2+^ (Table 4). The *K*_d_ values for the Pb^2+^removal in the presence of Na^+^, Ca^2+^, and Mg^2+^ in nano pure water (as a medium) are nearly the same as the *K*_d_ values for the assessment without the counterions. This contrasts with the case of the ZnS aerogel, where the *K*_d_ values drop by an order of magnitude for Mg^2+^ and Ca^2+^, although the singly charged Na^+^ has minimal impact.^33^ Likewise, the layered potassium manganese sulfide also shows a decrease of an order of magnitude in *K*_d_ for Na^+^ and Ca^2+^, with the divalent cation having a more pronounced effect.^23^ Thus, the CaS aerogels are highly selective for Pb^2+^ relative to the hard Lewis basic cations that permeate water bodies, and no inhibition effect is observed for Ca^2+^.

**Table 4:** Distribution Coefficients for Pb^2+^ in the Presence of Competing Ions

**initial Pb** ^**2+**^ **conc. (mM)**	**competing ions and conc. (M)**	***K***_**d**_ **for Pb** ^**2+**^ **(mL/g)**	**removal (%)**	**previous report,** ^33^ ***K***_**d**_ **(mL/g)** ^a^	**previous report,** ^23^ ***K***_**d**_ **(mL/g)** ^a^
0.837	none	2.27 × 10^4^	0	2.35 × 10^4^	1.1–8.9 × 10^5^
0.831	Na^+^(1 M)	1.03 × 10^4^	91.17	1.11 × 10^4^	8.34 × 10^4^
0.843	Mg^2+^(1 M)	1.34 × 10^4^	93.06	2.91 × 10^3^	n/a
0.847	Ca^2+^ (1 M)	2.21 × 10^4^	95.67	5.58 × 10^3^	1.88 × 10^4^

a The previously reported *K*_d_ values were obtained for the ZnS aerogel^33^ (0.830 mM Pb^2+^ ion) and a layered potassium manganese sulfide^23^ (1.45 mM Pb^2+^ ion and a V/m ratio is 900).

## Discussion

### Synthesis and Composition of CaS Aerogels

For the synthesis of CaS nanocubes, the oleic acid and oleyl amine purportedly coordinate with Ca^2+^ ions of the calcium acetate to form Ca-oleate and Ca-oleylamine, followed by introduction of diphenylthiourea (DPTU, sulfur source) and heating to 320 °C under inert atmosphere.^36,38^ The PXRD matches the expected phase for CaS, and there is no evidence of crystalline CaCO_3_ (Figure 1); however, the FTIR does exhibit a characteristic peak at 873 cm^–1^ for carbonate (Figure S5) and carbonate is also evident in the XPS data of CaS NPs (Figure S6). When a hydroxide base (TMAOH) was used in the ligand exchange of oleate/oleylamine for 4-fluorothiophenolate (FTP), crystalline CaCO_3_ was noted in the PXRD (Figure S2) and TEM (Figure S3 and Table S1) attributed to CO_2_ absorption by Ca­(OH)_2_, leading us to switch to an amine base, which eliminated crystalline carbonate in the ligand-exchanged CaS NPs, although carbonate is still detected in the FTIR (Figure S5). The amorphous CaCO_3_, present in the as-prepared CaS NPs (and carried through each subsequent reaction to form the gel), is likely due to the well-known thermal decomposition of calcium acetate to form calcium carbonate and acetone. The amorphous CaCO_3_ was considered inconsequential until thermal analysis studies were conducted to assess the residual ligand content. The weight loss between 460 and 710 °C is characteristic of CO_2_ loss from calcium carbonate to form CaO, and the formation of CaO is verified by PXRD of the product from thermal treatment of a CaS aerogel at 800 °C in flowing argon. The quantification of CaO/CaS by Rietveld refinement of the PXRD data suggests 29.9 wt % CaO is formed, comparable to calculations from TGA (Calculation S6, 36.7 wt %). These data suggest that the “CaS” aerogel actually consists of ∼50 wt % CaCO_3_ (43.3 wt % from Rietveld analysis and 50.1 wt % from TGA) carried over from the original nanoparticle synthesis. With the goal of eliminating (or reducing) the amorphous CaCO_3_ content, the synthesis of CaS NPs was slightly modified by increasing the concentration of the sulfur source (DPTU) and the temperature in order to induce calcium acetate to react with sulfide before it can undergo decomposition to carbonate. This strategy was partially successful, leading to CaS aerogels with reduced carbonate contamination (∼15 wt %, see Figures S9 and S10). By comparing activity for Pb^2+^ uptake as a function of carbonate concentration, we are able to partially deconvolute the role of calcium carbonate vs calcium sulfide in Pb^2+^ exchange and sorption.

### Gel Formation Mechanism from Surface Chemistry

XPS results (Figure 5) demonstrate that CaS gelation proceeds through oxidative assembly of surface sulfide species, generating polysulfide (S–S) linkages that bridge neighboring nanoparticles into a three-dimensional network, despite the presence of significant CaCO_3_ impurity. Presumably, carbonate domains passivate a fraction of Ca^2+^ sites while leaving sufficient exposed sulfide to sustain network formation and subsequent ion-exchange or sorption reactions. Importantly, the identification of S–S bonding provides spectroscopic support for a gelation pathway driven by oxidative assembly (Scheme 1). This mechanism explains the ability of CaS nanoparticles to form extended networks with large surface areas and significant mesoporosity. Indeed, because the 4-fluorothiophenolate capping ligands are only present to control the kinetics of assembly, it should be possible to form gels without undergoing ligand exchange, as long as the particles are washed sufficiently to remove surface oleate/oleylamine and expose sulfide for oxidative assembly (Figure S12). In fact, direct oxidation of CaS NPs without prior ligand exchange also leads to CaS gelation via S–S linkages and formation of aerogels with comparable surface areas to those produced by the ligand-exchange route (151.1 vs 143.6 m^2^/g, Figures S13 and S14). This considerably simplifies the synthesis of CaS gels by eliminating the need to undergo ligand exchange, without compromising the textural, chemical, and compositional features.

### CaS Cation Exchange with Pb^**2**+^ Ions

The thermodynamics and kinetics of cation exchange in the CaS gel by the Pb^2+^ ion depend on four factors: (i) the acidity and basicity of ions present in the reaction, (ii) the difference between the solubility product constant, *K*_sp_, of the reactant (CaS) and product (PbS), (iii) the crystallite size of the gel components, surface area, and pore characteristics, and (iv) the presence of competing ions (Na^+^, Mg^2+^, and Ca^2+^).

The cation exchange of CaS to PbS is thermodynamically favorable as explained according to Pearson’s hard–soft acid–base (HSAB) theory. Ca^2+^ is a hard acid, while S^2–^ is a soft base. Ca^2+^ readily dissociates from S^2–^ in an aqueous solution. Since soft acids prefer binding with soft bases, Pb^2+^ forms a more stable compound with S^2–^ than Ca^2+^ does. The overall cation exchange reaction is shown in eq 1.

$$\mathrm{CaS}\left(s\right)+{\mathrm{Pb}}^{2+}\left(\mathrm{aq}\right)⇌\mathrm{PbS}\left(s\right)+{\mathrm{Ca}}^{2+}\left(\mathrm{aq}\right)$$ 1

where the reaction can be divided by two solubilization processes (eqs 2 and 3)­

$$\mathrm{CaS}(s)⇌{\mathrm{Ca}}^{2+}(\mathrm{aq})+S^{2-}(\mathrm{aq})\;K_{\mathrm{sp}}=8\times 10^{-6}$$ 2

$$\mathrm{PbS}(s)⇌{\mathrm{Pb}}^{2+}(\mathrm{aq})+S^{2-}(\mathrm{aq})\;K_{\mathrm{sp}}=3.2\times 10^{-33}$$ 3

The overall equilibrium constant for the cation exchange reaction is *K*_sp CaS_/*K*_sp PbS_ = 2.5 × 10^27^.^33,34^ As the equilibrium constant is enormously high, the exchange reaction from CaS to PbS is strongly favored thermodynamically. For this reason, the qualitative cation exchange of the CaS gel (50 wt % CaCO_3_) with Pb^2+^ was complete (Figures 7 and 8). The fact that no Ca was detected in the Pb^2+^ exchanged gel suggests that Pb^2+^ is exchanging for Ca^2+^ in CaS and for Ca^2+^ in CaCO_3_, even though the equilibrium constant for the cation exchange of the carbonate is much smaller than that for the sulfide (*K*_sp CaCO3_/*K*_sp PbCO3_ = 2.8 × 10^–9^/7.4 × 10^–14^ = 3.8 × 10^4^). Carbonate exchange is evident in Figure S11, where STEM-EDS and line-scan analysis show low sulfur intensity together with a strong and spatially persistent oxygen signal that correlates with Pb distribution, indicating that Pb is also coordinated in oxygen-rich environments, rather than exclusively as PbS. These compositional data are consistent with the formation of PbCO_3_ within the gel network as sulfide sites become limited. These data are consistent with previous studies showing that biogenic calcium carbonate and mesoporous carbonate-based nanocomposites are effective for Pb^2+^ remediation by cation exchange.^40,41^ The exchange is also kinetically fast (complete within a few minutes), attributed to the small crystallite size of the exchanging elements, the high, accessible surface area of the CaS aerogels, and the prevalence of mesopores.

The quantitative exchange of CaS aerogels (50 wt % CaCO_3_) was conducted with Pb^2+^ concentrations from 100 ppb to 20,000 ppm, and the performance metrics were compared to our previously published ZnS aerogels. For a 100 ppb Pb^2+^ solution, the CaS aerogel reduced the concentration to 5.4 ppb within 1 h (Figure 9a), whereas it took 16 h to drive the Pb^2+^ concentration below 15 ppb for the ZnS aerogel.^33^ The difference in the rate is attributed to the greater thermodynamic driving force for CaS vs ZnS, with ZnS having a *K*_sp_ value smaller than CaS by 20 orders of magnitude (ZnS *K*_sp_ = 2 × 10^–25^ vs CaS *K*_sp_ = 8 × 10^–6^), so that the equilibrium constant for the exchange of Pb^2+^ with ZnS is 6.3 × 10^7^. At 36 h (Table 1), the *K*_d_ value of the CaS aerogel (4.9 × 10^4^ mL/g) is ∼7 times greater than the *K*_d_ value of ZnS (7.27 × 10^3^) and the removal percentage of the CaS aerogel (98.1%) is also higher than that of the ZnS aerogel (87.3%). Given the large differences in the equilibrium constant for CaS vs ZnS, a greater performance difference might be expected but is likely mitigated by the high proportion of CaCO_3_ (∼50 wt %) in the CaS, which has a less favorable equilibrium constant than Pb^2+^ exchange with ZnS (3.8 × 10^4^ vs 6.3 × 10^7^).

The importance of the pore structure on exchange kinetics becomes evident when CaS aerogels are compared with ambiently dried CaS xerogels. While the xerogel exhibits lower surface areas due to pore shrinkage/collapse, they are still quite substantial (106 m^2^/g, Figure S8b); however, the pores are considerably smaller on average relative to aerogels (Figure S8c), which may limit diffusion. Still, the textural data alone does not explain the considerably poorer uptake of Pb^2+^ by the CaS gels, which is limited to ca. 40% of available Pb^2+^ ions in a 100 ppb solution, achieving saturation within 1 h. This is likely due to limited pore interconnectivity, accessibility, and diffusion pathways in the partially collapsed xerogel.^42^

For Pb^2+^ concentrations of 20–2000 ppm (Figure 10 and Table 2), *K*_d_ values and removal percentages remain high (*K*_d_ > 10^4^, >95% removal) for CaS aerogels (∼50 wt % CaCO_3_). Moreover, the presence of 1 M competing ions (Na^+^ and Mg^2+^) or the product ions (Ca^2+^) has a minimal effect on CaS aerogel exchange with Pb^2+^, as demonstrated by an absence of significant changes in the *K*_d_ values and the removal percentage (Table 4). The high selectivity of CaS aerogels toward Pb^2+^ is due to the hardness of Ca^2+^ ions and the consequent dissolution of Ca^2+^ in water. Since Na_2_S is more soluble than CaS, and MgS is not stable in water, there is no driving force to exchange with CaS as there is for Pb^2+^. Thus, the modest inhibition noted for Na^+^ and Mg^2+^ can be attributed to charge screening. Since Ca^2+^ is the product of exchange, a high concentration of free Ca^2+^ might be expected to inhibit Pb^2+^ exchange, but the high solubility product of CaS is such that there is no inhibition effect when 1 M Ca^2+^ is present. In the context of competing ions, a comparison to our prior ZnS aerogel as well as to a highly selective layered potassium manganese sulfide system is illustrative (Table 4). While both systems show excellent selectivity to Pb^2+^, *K*_d_ values decrease by an order of magnitude in the presence of divalent cations (Mg^2+^, Ca^2+^).

The maximum removal capacity of CaS aerogels (∼50 wt % CaCO_3_) is 17.1 mmol Pb^2+^/1 g of CaS aerogels (3538 mg/g), far greater than predicted based on cation exchange alone (11.9 mmol Pb^2+^/g of CaS aerogels, Calculation S8). The measured Pb^2+^ uptake capacity reflects the combined contributions of sulfide-driven precipitation (PbS formation upon cation exchange), S–S-mediated network reactivity (sorption), and carbonate-assisted Pb^2+^ capture (PbCO_3_ formation) rather than the stoichiometric capacity of CaS alone. This capacity is particularly impressive given the significant amount of amorphous carbonate present in the samples (∼50%), which has a relatively low affinity for Pb^2+^. Indeed, reduction of the calcium carbonate phase from ∼50 to ∼15 wt % results in a substantial increase in capacity to 22.51 mmol/g of the CaS gel (vs 13.26 mmol/g, theoretical). To the best of our knowledge, this capacity is greater than that reported for any other chalcogenide aerogel systems, including K–Co–Mo-S_*x*_ chalcogels (5.53 mmol Pb^2+^/g) (Table 5), and our prior work on ZnS (14 mmol Pb^2+^/g).^29,33^

**Table 5:** Comparison of Pb^2+^ Sorption Performance of CaS Aerogels with Representative Aerogel and Chalcogel Sorbent Systems, Including Uptake Capacity, Sorption Kinetics, Selectivity in the Presence of Competing Ions, and Stability or Reusability^a^

**sorbent (aerogel/chalcogel)**	**Pb** ^**2+**^ **removal capacity**	**kinetics (equilibrium time)**	**selectivity/competitive ions**	**stability/reuse**	**reference**
**CaS aerogel (∼50 wt % CaCO**_**3**_ **)**	**17.1 mmol g^–1^ (3543mg g^–1^)**	<1 h	high selectivity vs Na^+^, Ca^2+^, Mg^2+^	stable during uptake	**this work**
**CaS aerogel (∼15 wt % CaCO**_**3**_ **)**	**22.5 mmol g^–1^ (4664mg g^–1^)**	<1 h	high selectivity vs Na^+^, Ca^2+^, Mg^2+^	stable during uptake	**this work**
ZnS nanoparticle aerogel	14.2 mmol g^–1^ (2940 mg g^–1^)	2–6 h	high selectivity vs Na^+^, K^+^, Ca^2+^, Mg^2+^	precipitation-dominated; limited reuse	^33^
K–Co–Mo–S_*x*_ (KCMS) chalcogel	5.53 mmol g^–1^ (1146 mg g^–1^)	<1 h	Pb^2+^ > Ag^+^ > Hg^2+^ > Cu^2+^ ≫ Cd^2+^, Ni^2+^; tested in river water	framework stable; regeneration not shown	^29^
cellulose-based carbon aerogel	1.16 mmol g^–1^ (240 mg g^–1^)	4–8 h	minimal effect of Na^+^, K^+^, Ca^2+^	reusable by acid wash	^43^
reduced graphene oxide aerogel	0.28 mmol g^–1^ (58 mg g^–1^)	3–6 h	moderate selectivity in mixed-ion systems	reusable	^44^
DTPA-functionalized GO aerogel	1.5 mmol g^–1^ (310 mg g^–1^)	1–2 h	selective Pb^2+^ over Cu^2+^, Cd^2+^	reusable (chelation-based)	^45^
calcium alginate aerogel	1.9 mmol g^–1^ (390 mg g^–1^)	1–3 h	Pb^2+^ > Cd^2+^, Cu^2+^	reusable	^46^
alginate/GO composite aerogel	1.8 mmol g^–1^ (368 mg g^–1^)	∼40 min	Pb^2+^/Cd^2+^/Cu^2+^ mixed system	regenerable	^47^
alginate/biochar aerogel spheres	3.2 mmol g^–1^ (664 mg g^–1^)	∼2 h	tested in complex wastewater	reusable	^48^

a Reported capacities are provided in both mmol g^–1^ and mg g^–1^.

While the sorption performance for lead is exceptional, we emphasize that this system is best viewed as a high-capacity single-use, or limited-use, sorbent analogous to sulfide-based stabilization strategies commonly employed in hazardous waste treatment. In such applications, the goal is not repeated regeneration but rather rapid and irreversible capture of toxic metals followed by safe containment of the resulting metal sulfide solids. Because the Pb^2+^ removal by CaS aerogels proceeds predominantly through precipitation-driven PbS formation driven by a strong thermodynamic potential, simple regeneration via aqueous elution is not expected to be practical. As shown in Table 5, carbon- and/or alginate-based aerogels are demonstrated to uptake Pb^2+^ reversibly, enabling regeneration or reuse; however, capacities are much lower (0.28–3.2 mmol Pb^2+^/g) than those of the chalcogenide-based systems, owing to a weaker thermodynamic driving force. Although the capacities for CaS aerogels are high, CaS is susceptible to hydrolysis and carbonation under aqueous and ambient conditions. While this does not appear to greatly impede the gel performance in the short term, long-term environmental aging studies are needed to determine whether this novel sorbent will remain active on the shelf. Finally, while it was demonstrated to be selective to Pb^2+^ in the presence of common ions found in natural water sources, future work is needed to assess the efficacy in real water sources, where organic matter and a broader range of dissolved species are present.

## Conclusions

The current study shows that previously demonstrated methodologies for the formation of II–VI and IV–VI quantum dot aerogels via oxidative assembly can be extended to the more ionic alkaline earth sulfides (specifically, CaS) and utilized for remediation of Pb^2+^ ions. The CaS aerogel was specifically designed for selective sorption of soft Lewis acidic heavy metals, driven by the hard Lewis acidity of the Ca^2+^ that drives dissolution in water and the soft Lewis basic sulfide anions that form the framework of the gel. The small size of the primary crystallites and the porous architecture deliver a high surface area framework for rapid cation exchange and chemisorption. However, the CaS gel incorporates amorphous carbonate impurities that carry over from the original CaS nanoparticle synthesis, making up approximately 50 wt % of the aerogel samples. Nevertheless, treatment with Pb^2+^ solutions results in rapid (minutes) and complete exchange of all Ca^2+^ in the system, including from amorphous calcium carbonate, even though the equilibrium constant is far smaller than that for CaS (*K*_eq_ = 3.8 × 10^4^ vs 2.5 × 10^27^). At a low Pb^2+^ concentration (100 ppb), the CaS aerogel removed 97% (5.4 ppb) of Pb^2+^ within 1 h of exchange (below the EPA action limit of 15 ppb) and removed down to 1.9 ppb after 36 h. Removal efficiencies were similar for higher concentrations of lead (20–2000 ppm), even in the presence of 1 M competing ions (Na^+^, Mg^2+^, Ca^2+^), and a quantitative study with 20,000 ppm shows that the capacity of Pb^2+^ ion removal is 17.1 mmol Pb^2+^/g. This capacity is greater than that predicted based on cation exchange alone (11.9 mmol/g), indicative of Pb^2+^ chemisorption on the sulfide framework (and disulfide linkers) of the gel. Moreover, the capacity can be further increased by modifying the CaS nanoparticle synthesis to limit CaCO_3_ to 15 wt % CaCO_3_ (22.5 mmol/g of CaS vs 13.3 mmol/g theoretical). The interconnected pore structure of the aerogels is critical for removal efficiency as CaS xerogels prepared by ambient pressure drying became saturated after absorbing only 40% of Pb^2+^ ions in a 100 ppb solution, attributed to pore shrinkage, collapse, and loss of pore interconnectivity. Notably, the CaS aerogel supersedes the previously studied ZnS aerogels for Pb^2+^ remediation in terms of rate, selectivity, *K*_d_, and capacity, without delivering toxic Zn^2+^ to the environment. Indeed, the performance exceeds that of some of the most active and selective Pb^2+^ exchangers reported. Future work is focused on long-term studies of CaS aerogel stability with respect to carbonation and testing in simulated and real water systems.

## Supplementary Material



## Notes

TEM image of CaS NPs and the particle size histogram; PXRD, HAADF STEM, EDS mapping of CaS NPs before and after ligand exchange with FTP in the presence of TMAOH; NMR and FTIR of CaS NPs, FTP, FTP-capped CaS NPs, and CaS aerogels (∼50 wt % CaCO_3_); HAADF-STEM images of CaS gels and EDS composition/line scans; BET surface areas and pore size distributions for a CaS aerogel (∼15 wt % CaCO_3_) and xerogel; TGA and HAADF-STEM EDS mapping for a CaS aerogel (∼15 wt % CaCO_3_); HAADF-STEM image of a PbS gel prepared by ion exchange of a CaS aerogel (∼50 wt % CaCO_3_); scheme for gelation without ligand-exchange; HAADF-STEM, EDS composition, nitrogen physisorption isotherms, and XPS of the resultant gels (Figures); compositional data for CaS gels prepared from particles treated with TMAOH vs TEA (Tables); remediation studies with 0.1, 20, 200, 2000, and 20,000 ppm Pb^2+^ solution with CaS aerogels; remediation studies with 0.1 ppm Pb^2+^ solution with a CaS xerogel; theoretical exchange capacity of CaS aerogels; wt % of CaS and CaCO_3_ in CaS aerogels from TGA data; and Rietveld refinement data for a CaS aerogel heated to 800 °C in flowing Ar (Calculations) (PDF)
