# Supplementary material for: Selective Sorbent Design: CaS Aerogel for Rapid Remediation of Aqueous Pb (II)
Source: Chem Mater. 2026 Mar 30;38(7):3361–73. doi: 10.1021/acs.chemmater.5c03082 (PMC13084990; doi:10.1021/acs.chemmater.5c03082)
Supplement: Supplementary file 1 [file cm5c03082_si_001.pdf]

Selective sorbent design: CaS aerogel for rapid remediation of aqueous Pb (II)

Md Masudur Rhaman and Stephanie L. Brock*

Department of Chemistry, Wayne State University, 5101 Cass Avenue, Detroit, Michigan 48202, United States.

*Email: sbrock@chem.wayne.edu

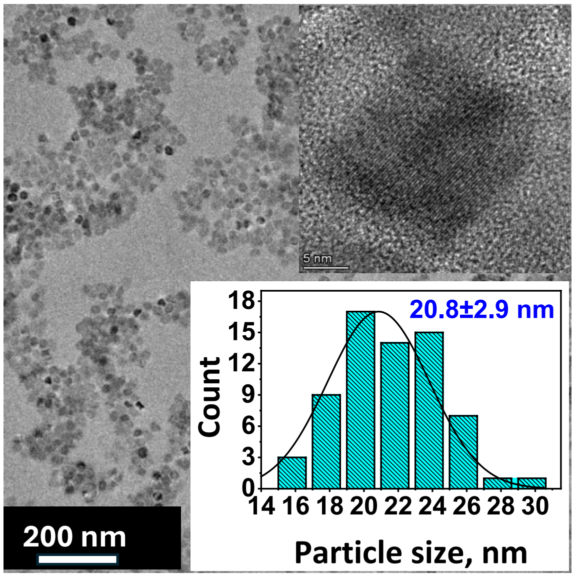

Figure S1. TEM image of CaS nanocubes. The top inset shows a single cube (scale bar = 5 nm) and the bottom inset shows the particle size distribution (size corresponds to the *diagonal body* of a nanocube)

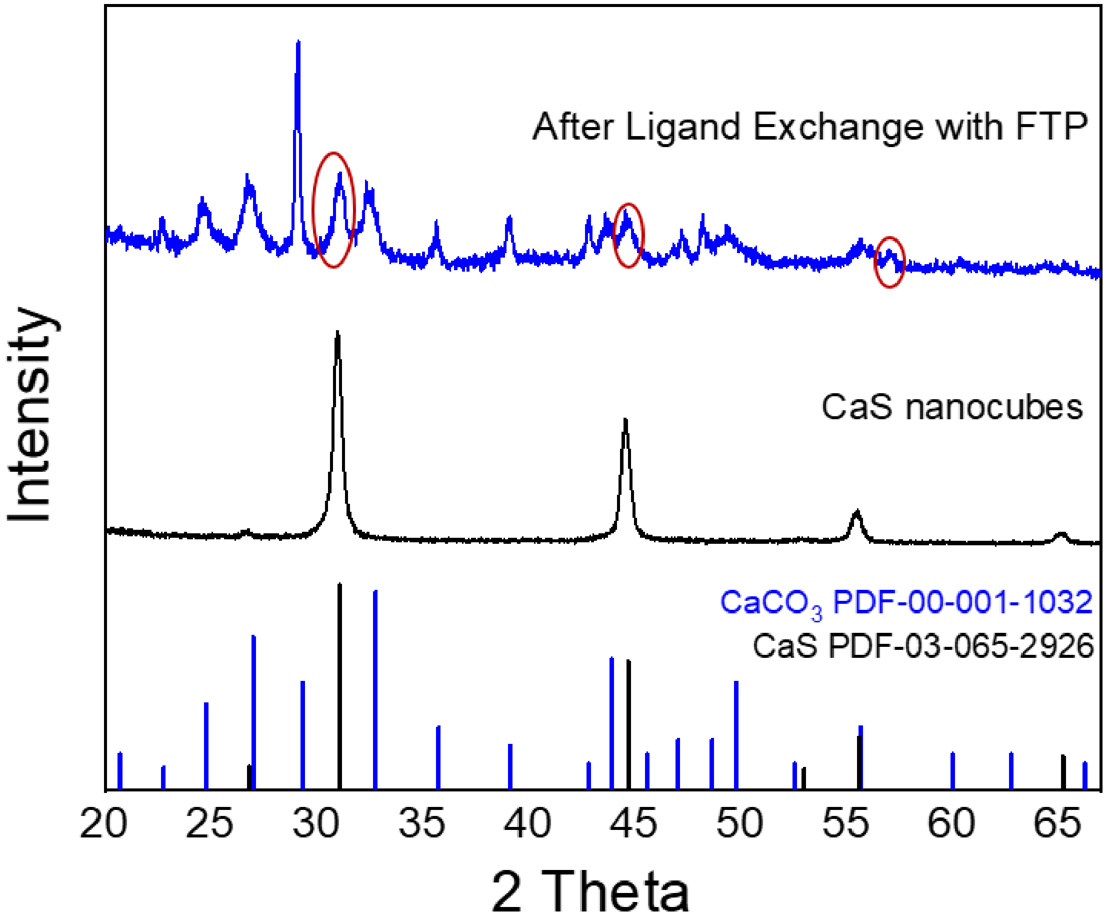

Figure S2. PXRD of CaS nanoparticle before (lower trace) and after (upper trace) ligand exchange with FTP in the presence of TMAOH along with reference stick diagrams for $CaCO_3$ and CaS. The red circles indicate the presence of the CaS phase after ligand exchange in the upper trace, whereas the remaining peaks are attributed to $CaCO_3$.

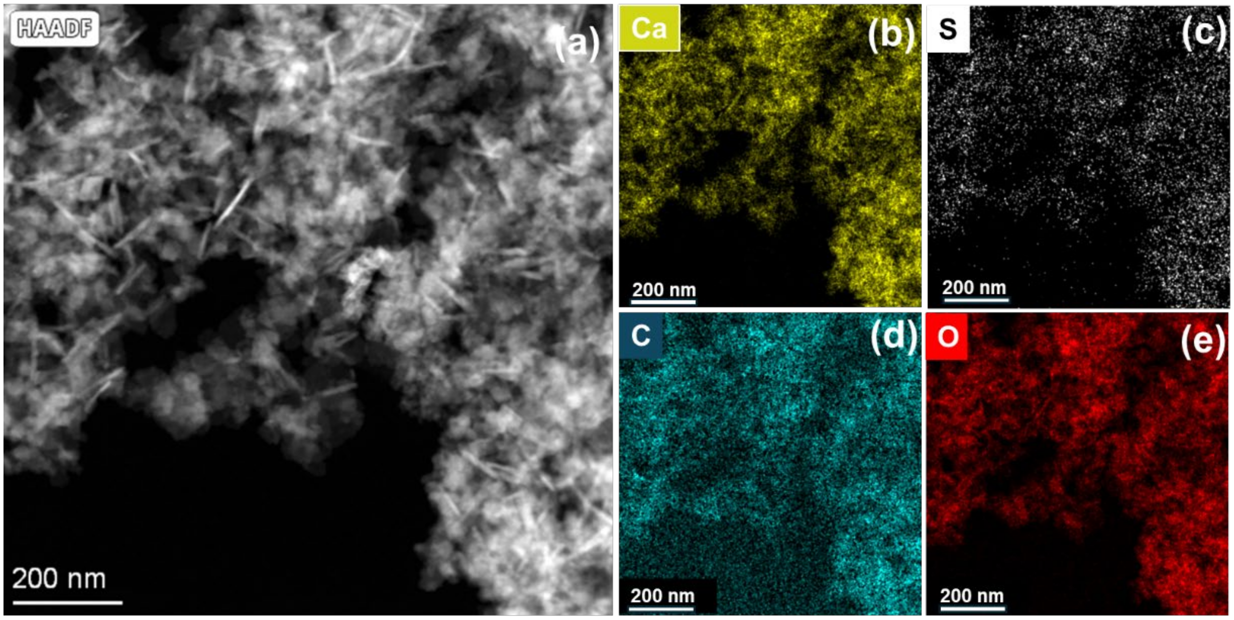

Figure S3. HAADF STEM EDS mapping images of a CaS gel synthesized in the presence of TMAOH (a) HAADF image, EDS mapping of (b) Ca (yellow), (c) S (white), (d) C (blue) and (e) O (red).

Table S1. Composition data from STEM-EDS of a CaS gel synthesized in the presence of TMAOH. Sulfur is barely detected, whereas significant concentrations of C, O and Ca are noted, consistent with $CaCO_3$ formation.

Element	Atomic Fraction (%)	Atomic Error (%)
C	52.34	3.77
N	1.65	0.35
O	35.20	4.59
S	0.67	0.13
Ca	10.15	1.41

Ligand exchange of oleate-capped ligand with 4 fluorothiophenol in the presence of TEA

Figure S4(a) shows the ^{1}H NMR of oleate-capped CaS NPs (which may also include oleylamine). A broad singlet peak at 5.48 ppm (circled in red) is attributed to the presence of vinyl protons associated with oleic acid/oleylamine. In **Figure S4(b),** the ^{1}H NMR of pure 4-fluorothiophenol in d-ethanol is shown, where the two peaks in the blue circle show the complex multiplicity associated with the presence of a monosubstituted benzene ring with a fluorine (-F) due to ^{19}F-^{1}H coupling. In addition, the proton on the -SH group in FTP is also evident as a broad singlet peak (green circle) at 4.18 ppm (inset). **Figure S4(c)** shows the FTP-exchanged CaS nanocubes. The absence of vinyl peaks at 5.48 ppm suggests most of the oleate/oleylamine has been successfully removed. In addition, the appearance of complex multiplicity peaks and absence of the -SH proton at 4.18 ppm suggests introduction of FTP as a thiolate. The down-field shift (relative to FTP in d-ethanol) of the complex multiplicity peaks after ligand exchange is consistent with thiol group deprotonation and surface binding to the CaS nanoparticles; the coordination of deprotonated thiol with Ca^{2+} de-shields the benzene ring due to withdrawing of electron density by S.

Successful ligand exchange is also supported by Fourier transform infrared spectroscopy (FTIR) as shown in **Figure S5**. The bands in oleate-capped CaS NPs at 2921 and 2847 cm^{-1} are assigned to the asymmetric and symmetric -C-H stretching vibrations of $-CH_2-$ groups in oleic acid (oleylamine), which become less intense (2921 and 2852 cm^{-1}) when CaS NPs are capped with 4-fluorothiophenol (FTP). In addition, new bands at 1225, 1155, and 1045 cm^{-1} are attributed to the -C-F stretching vibration in FTP.

After oxidative assembly of FTP-capped CaS NPs, the FTIR of the CaS gel doesn't show any significant bands for -C-F stretching peaks (**Figure S5**), suggesting most or all the FTP has been removed in the process. However, the magnified figure in the rectangle shows the presence of the asymmetric and symmetric C-H stretching vibrations of $-CH_2-$ groups at 2923 and 2852 cm^{-1}. These two peaks indicate some residual ligands remain on the CaS aerogel.

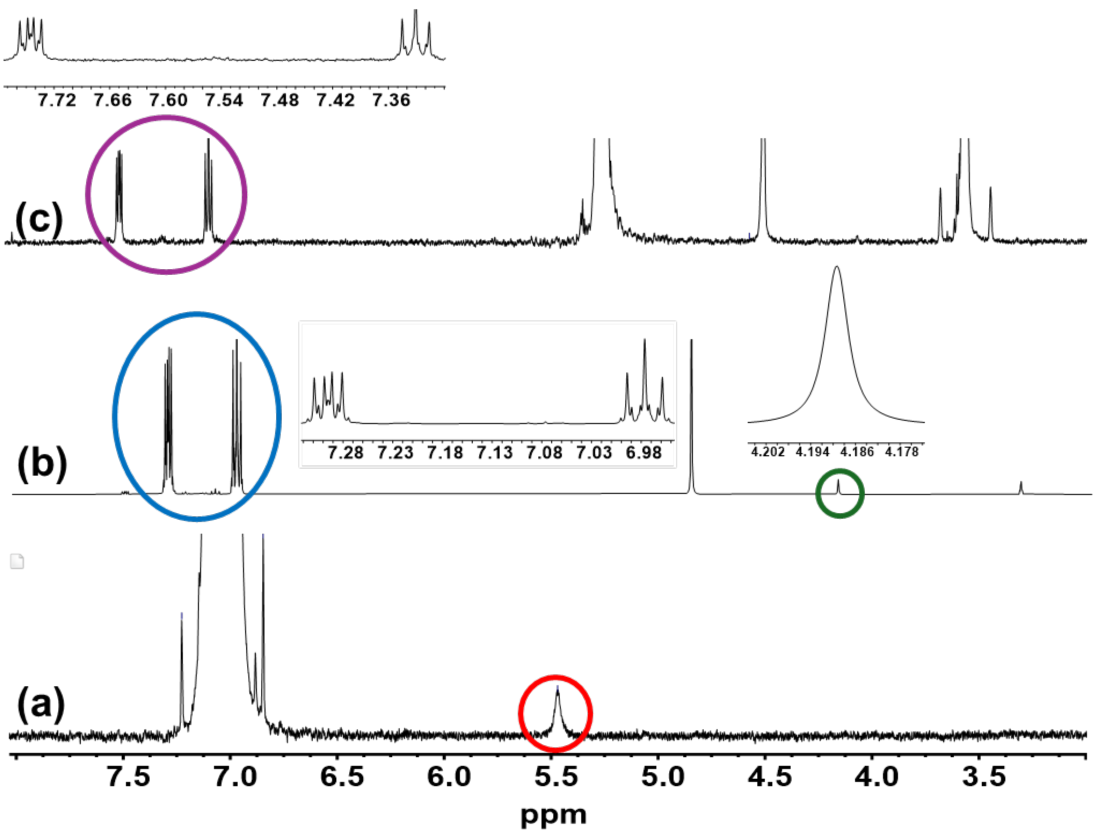

Figure S4. ^{1}HNMR of (a) CaS NPs capped with oleate and oleyl amine in d-toluene; (b) FTP in d-methanol, (c) CaS NPs capped with FTP in d-methanol. The red circle in (a) corresponds to vinylic protons attributed to oleate/oleylamine capping groups. The green circle (and expanded axis) in (b) corresponds to the thiol proton in FTP, whereas the blue circle (and expanded axis) show complex multiplets associated with ^{1}H-^{19}F NMR coupling of benzyl protons with the ortho/meta fluorine group. The purple circle (and expanded axis) in (c) corresponds to the ^{1}H-^{19}F NMR coupling as in (b), but the peaks are shifted downfield due to binding to the surface of CaS.

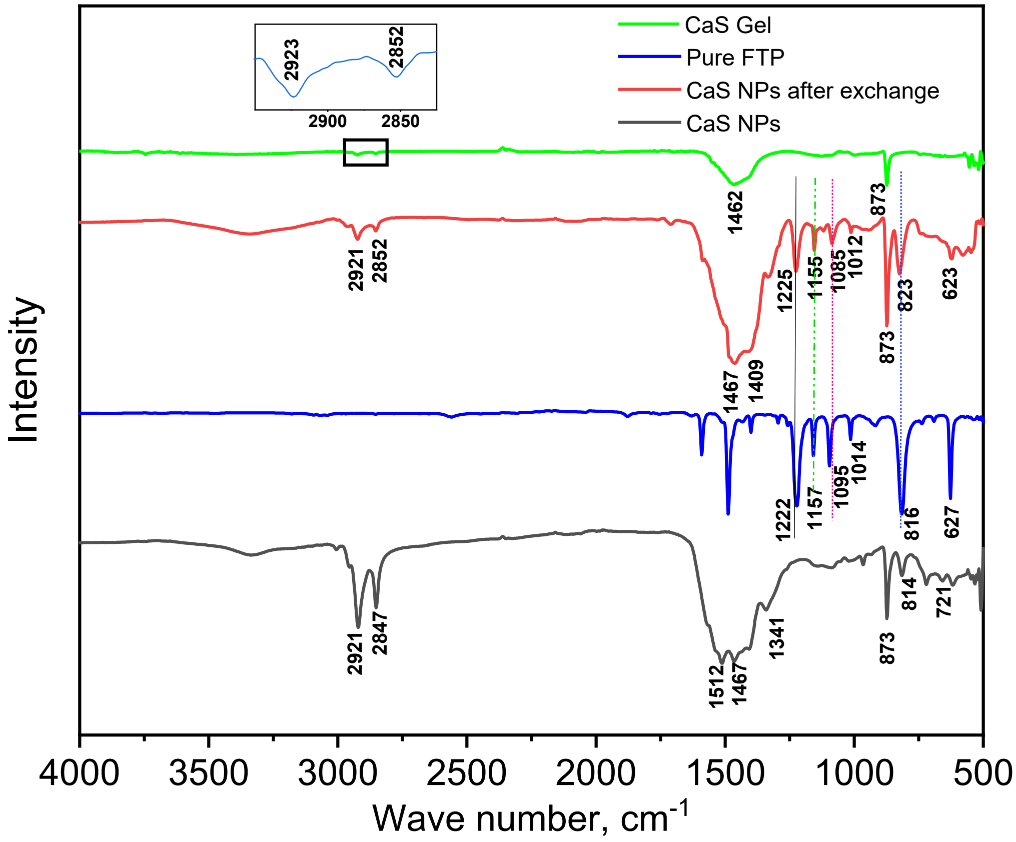

Figure S5. FTIR of CaS NPs, pure FTP, CaS NPs capped with FTP, and the CaS gel. The inset corresponds to the rectangular area noted in the CaS gel.

Table S2. STEM-EDS of a CaS gel synthesized in the presence of TEA

Element	Atomic Fraction (%)	Atomic Error (%)
F	**0.51**	0.33
S	45.15	5.51
Ca	55.34	5.44

Interpretation of XPS data acquired on as-synthesized CaS NPs

The XPS data of as-synthesized CaS NPs is shown in **Figure S6**. The Ca 2p region consists of multiple components, including a Ca–S (CaS) contribution and a higher binding-energy Ca environment that is consistent with carbonate-coordinated Ca ($CaCO_3$-like). A carbonate (CO_3^{2-}) component (near the ~289 eV region) is also found in the C 1s, and the O 1s spectrum contains a strong component assigned to carbonate oxygen, supporting the conclusion that CO_3^{2-} is present at the surface (i.e., a $CaCO_3$-like layer/patches or carbonate-containing surface species). In addition to carbonate formation, the presence of higher binding-energy (163.9 eV) components in the S 2p spectrum suggests elemental S^0 is forming upon surface oxidation, along with the characteristic sulfide (S^{2-}) doublet attributed to CaS. The presence of these higher-BE features indicates that a fraction of surface sulfide has undergone partial oxidation, consistent with a thin oxidized shell forming during handling/aging. The N 1s signal is consistent with the presence of oleylamine-derived nitrogen (or weak surface-bound amines).

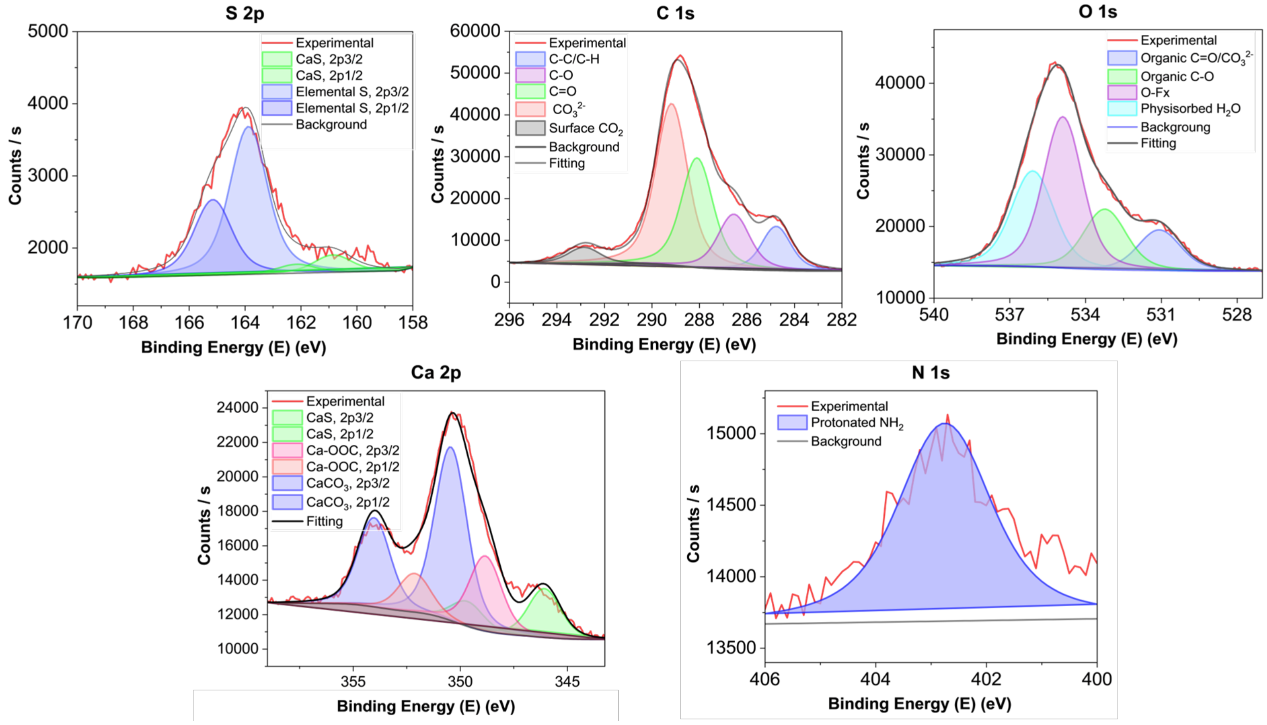

Figure S6. X-ray photoelectron spectroscopy (XPS) analysis of oleic acid/oleylamine–capped CaS nanoparticles synthesized from $Ca(CH_3COO)_2 \cdot H_2O$ and diphenylthiourea. High-resolution spectra of S 2p, C 1s, O 1s, Ca 2p, and N 1s regions are shown. Solid red lines represent experimental data, black lines denote fitted envelopes, and shaded components correspond to individual chemical states. The dominance of organic carbon signals confirms ligand-stabilized CaS nanoparticles prior to oxidative gelation, and the calcium, oxygen and carbon speciation is consistent with calcium carbonate formation as a byproduct.

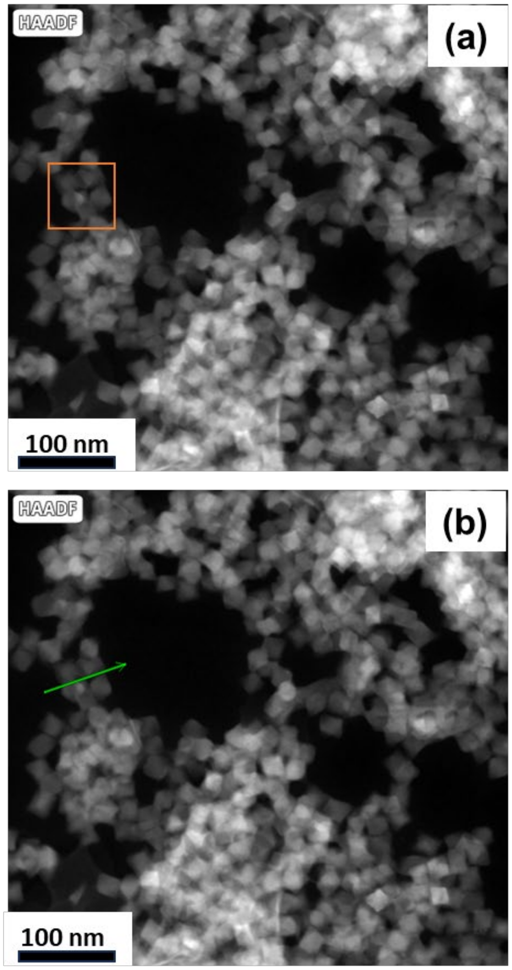

Element	Atomic Fraction (%)	Atomic Error (%)
C	76.82	2.24
O	10.35	1.94
F	0.59	0.17
S	5	0.94
Ca	7.24	0.98

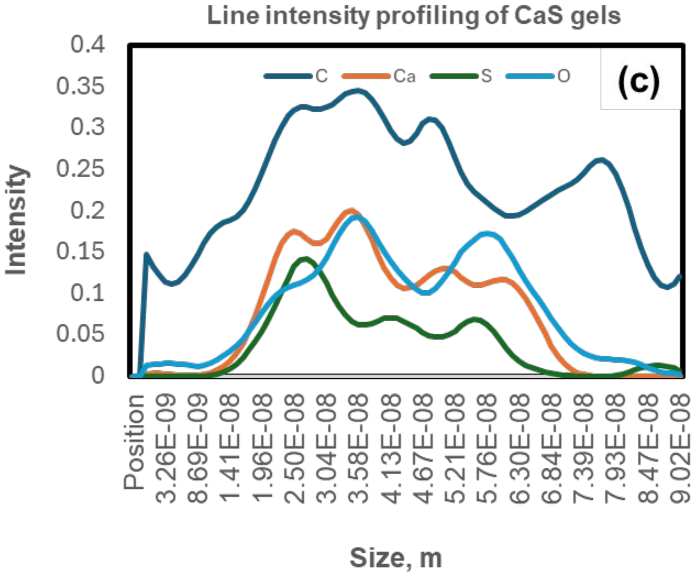

Figure S7. (a,b) HAADF-STEM images of the CaS nanoparticle gel showing a highly interconnected three-dimensional network formed by nanoscale primary particles (scale bars: 100 nm). The orange box in (a) and arrow in (b) indicate representative regions used for compositional analysis. (c) Line-intensity profiles extracted along the indicated path (green line) show the spatial distribution of C, Ca, S, and O across the gel network. The accompanying EDS quantification table summarizes the average atomic fractions and associated errors for the detected elements.

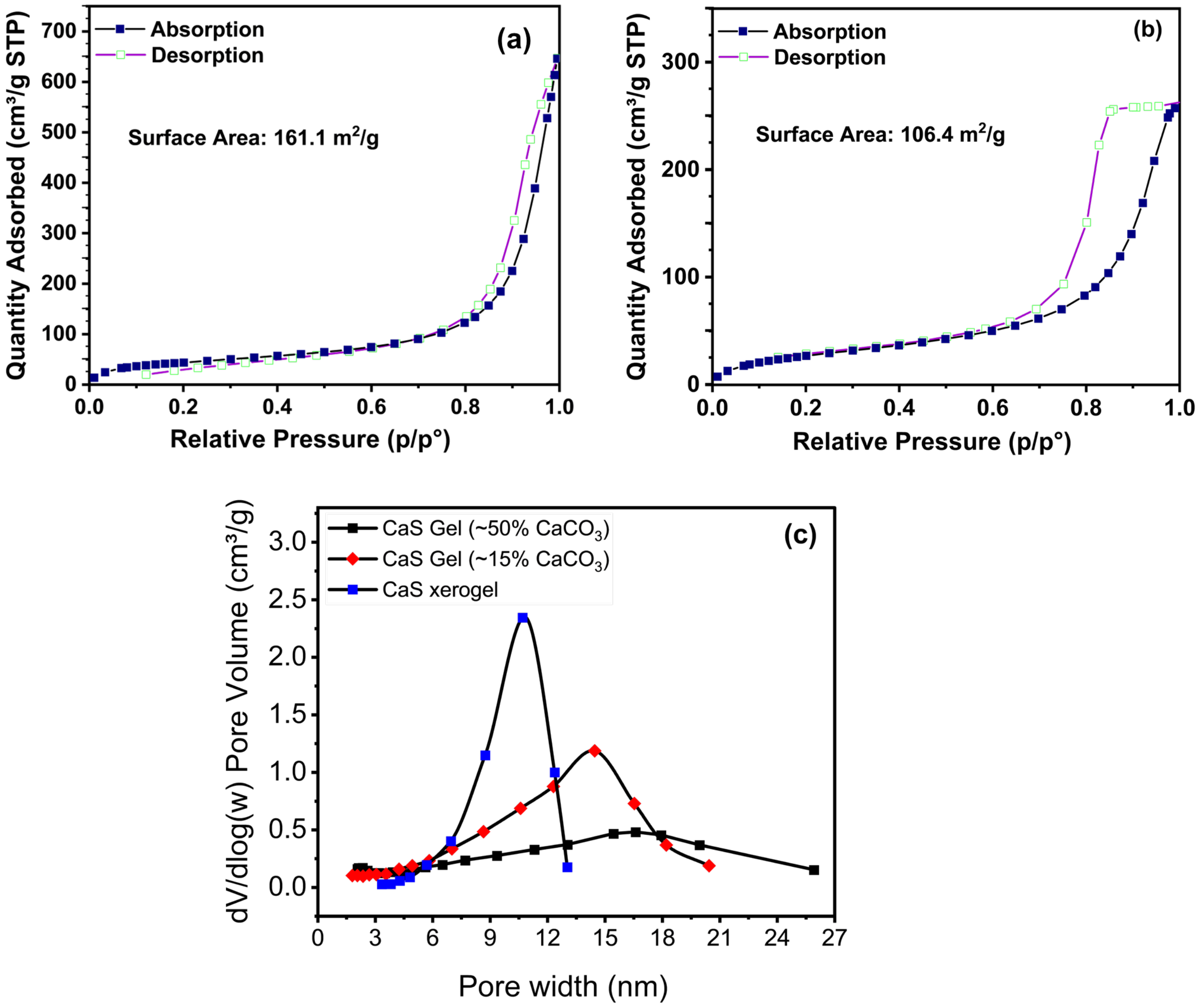

Figure S8. Textural characterization of CaS aerogels with varying carbonate content and a xerogel (a) N_2 adsorption–desorption isotherm of the CaS gel containing ~15% $CaCO_3$ (BET surface area: 161.1 m² g⁻¹). (b) N_2 adsorption–desorption isotherm of the corresponding CaS xerogel, showing a reduced surface area (106.4 m² g⁻¹). (c) BJH pore size distributions of CaS gels (~50% and ~15% $CaCO_3$) and the CaS xerogel, indicating comparable mesopore sizes (~3–25 nm) but significant differences in pore volume.

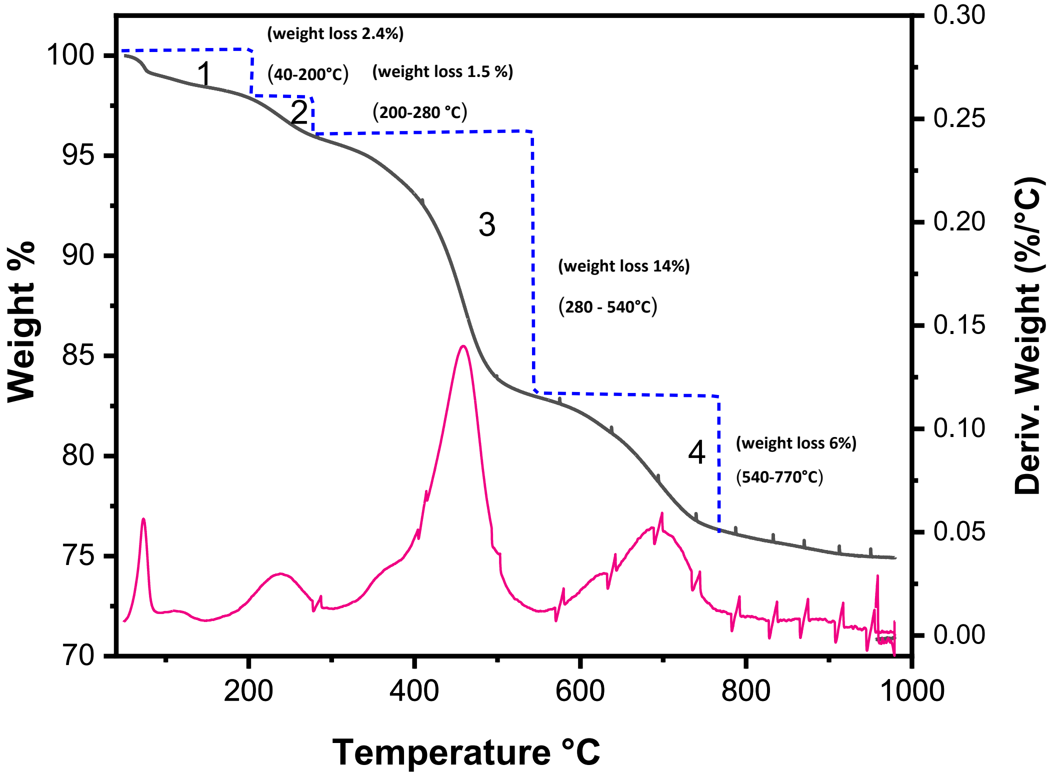

Figure S9. Thermogravimetric analysis of CaS aerogel with ~15% $CaCO_3$ where the weight loss with temperature and the derivative weight with temperature shown in black and pink, respectively and the stages of weight loss are numbered 1, 2, 3 and 4. The weight loss in stage 1 (40°C-200°C), 2 (200-280°C), 3 (280-540°C) and 4 (540-770°C) are 2.4%, 1.5%, 14% and 6%, respectively.

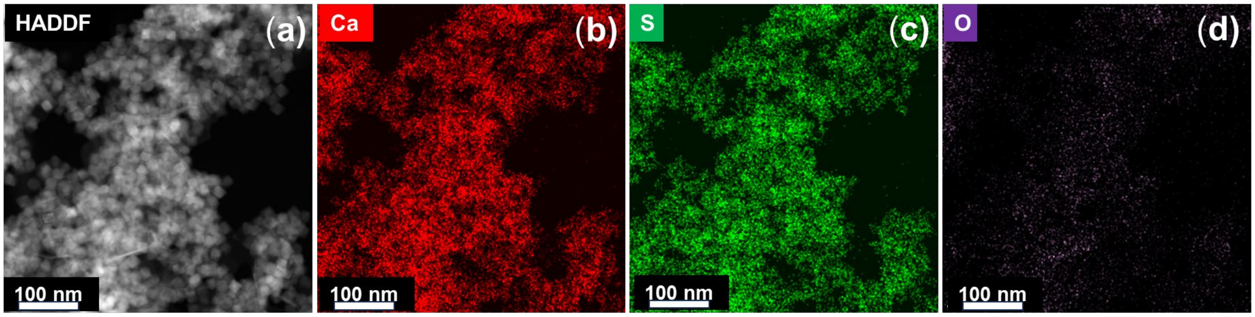

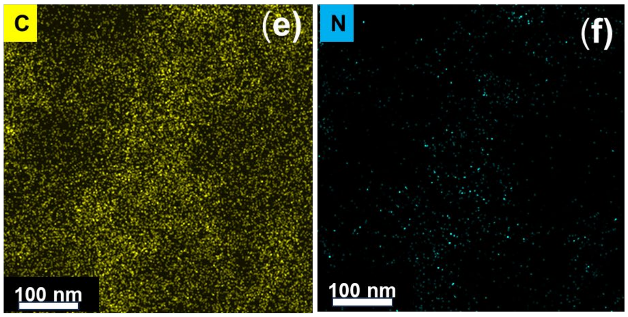

Element	Atomic Fraction (%)	Atomic Error (%)
C	52.45	2.49
N	0.44	0.29
O	10.96	2.17
S	17.33	2.49
Ca	18.82	2.17

Figure S10. HAADF-STEM image (a) and corresponding STEM-EDS elemental maps (b–f) of Ca, S, O, C, and N for the CaS gel containing ~15% $CaCO_3$, showing a highly interconnected nanoparticle network with homogeneous Ca and S distribution. Scale bars: 100 nm.

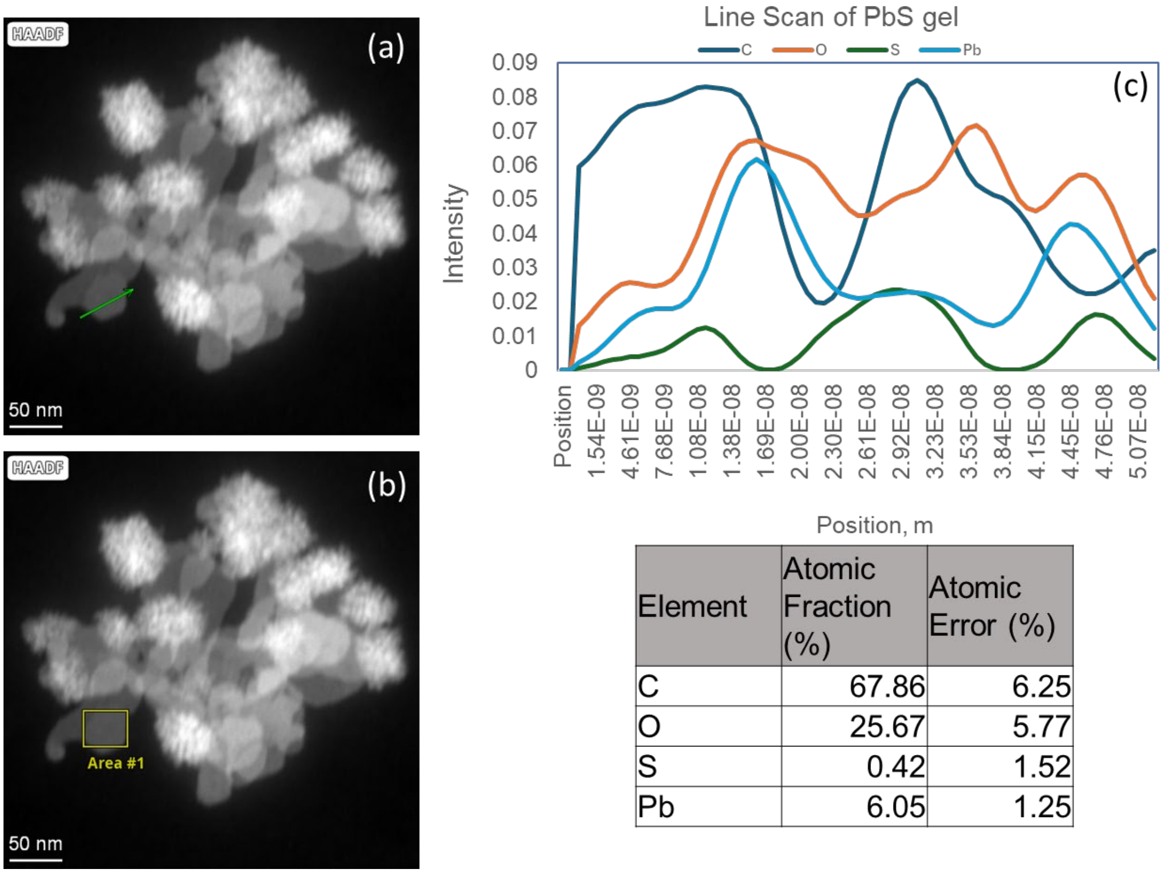

Element	Atomic Fraction (%)	Atomic Error (%)
C	67.86	6.25
O	25.67	5.77
S	0.42	1.52
Pb	6.05	1.25

Figure S11. HAADF-STEM images of the PbS gel showing (a) the line-scan path used for elemental analysis and (b) the selected area (Area #1) for compositional quantification. The corresponding EDS line scan (c) reveals the spatial distribution of C, O, S, and Pb across the gel network and the table inset shows the atomic fractions of C, O, S and Pb in Area #1. Scale bars: 50 nm.

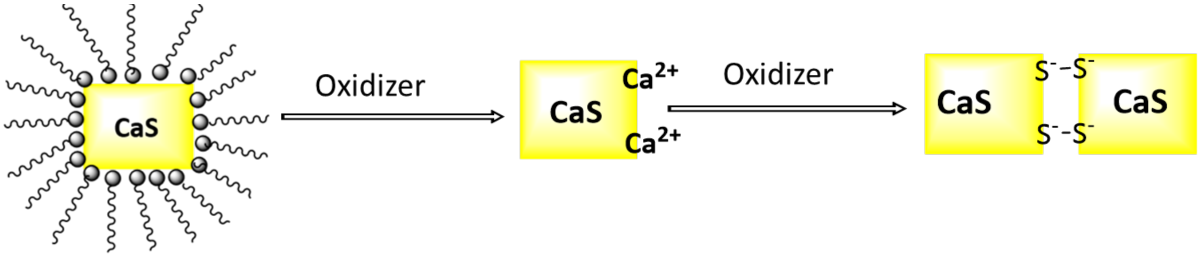

Figure S12. Schematic illustration of CaS nanoparticle gel formation via oxidative coupling without ligand exchange steps with FTP and TEA. Oleate-capped CaS nanoparticles are initially stabilized by surface ligands.

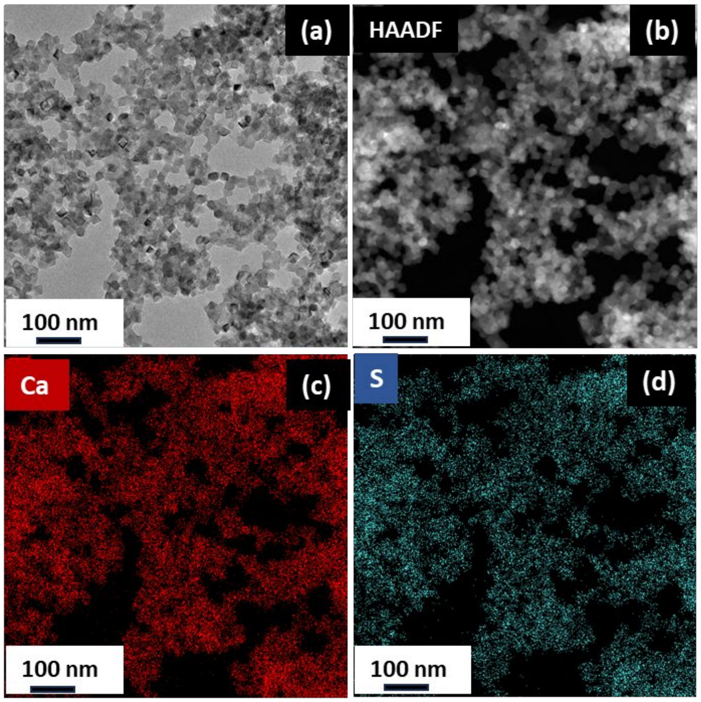

Element	Atomic Fraction (%)	Atomic Error (%)
S	**45.78**	3.31
Ca	**54.22**	3.31

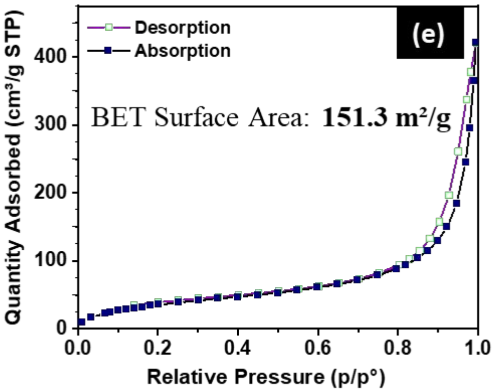

Figure S13. Structural, compositional, and textural characterization of the CaS nanoparticle aerogel. (a) Bright-field TEM image showing nanoscale CaS primary particles assembled into an extended network. (b) HAADF-STEM image highlighting the three-dimensional porous architecture of the aerogel. (c,d) STEM-EDS elemental maps of Ca and S, respectively, demonstrating homogeneous spatial distribution throughout the network. (e) N_2 adsorption–desorption isotherm of the CaS aerogel, indicating a mesoporous structure with high surface area. The accompanying EDS quantification table summarizes the atomic fractions and associated errors for Ca and S. Scale bars: 100 nm.

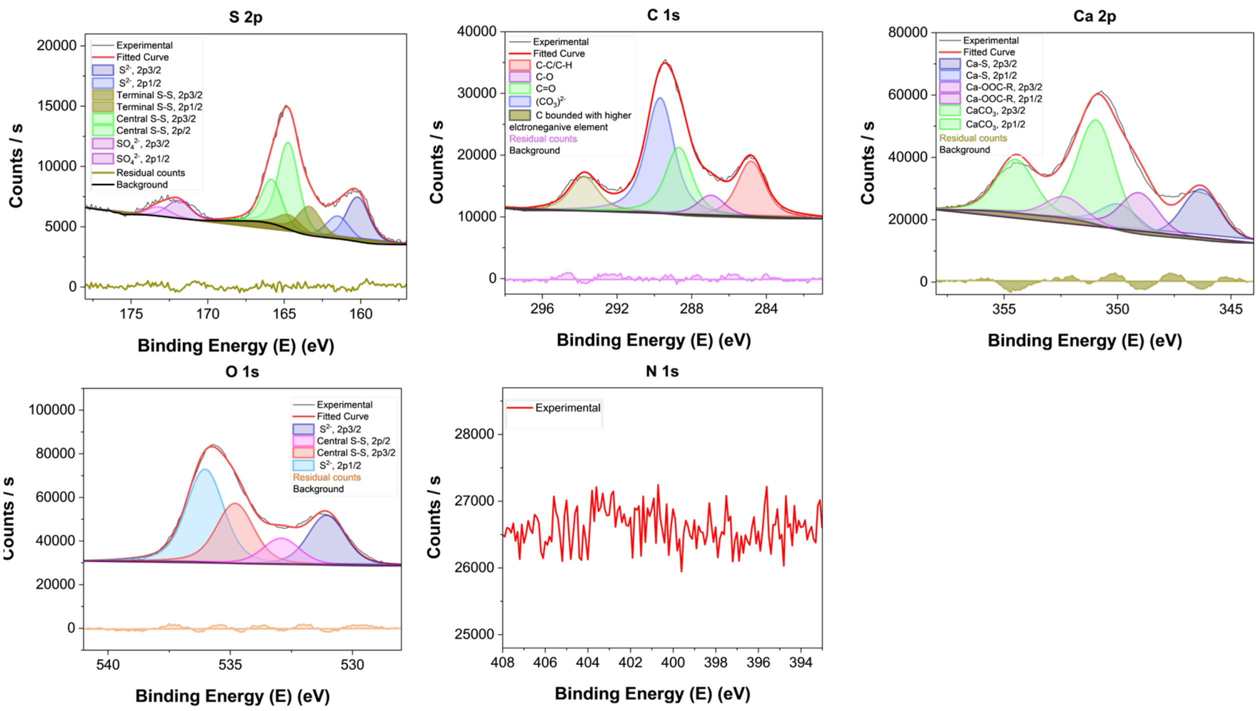

Figure S14. X-ray photoelectron spectroscopy (XPS) analysis of CaS nanoparticle gels prepared without ligand exchange, where gelation is induced solely by oxidative treatment with tetranitromethane (TNM). High-resolution spectra are shown for S 2p, C 1s, Ca 2p, O 1s, and N 1s. The S 2p spectrum reveals sulfide (S^{2-}) and polysulfide (S–S) species, indicating interparticle oxidative coupling. The Ca 2p and O 1s spectra show contributions from both CaS and surface $CaCO_3$ environments. Solid black lines represent experimental data, red lines correspond to fitted envelopes, and shaded components indicate individual chemical states.

Calculation S1: Removal of 100 ppb Pb^{2+} from 10.55±0.14 g (~10 mL) of solution with 10.15±0.32 mg of CaS gel

Experiment 1					
	**Weight (g)**	**Volume (mL)**	**Time (hours)**	**Ca^{2+} Conc. (ppb)**	**Pb^{2+} Conc. (ppb)**
CaS Aerogel	10.75		0	0	100.56
			0.17	2.53	55.79
			0.33	5.61	39.69
Pb^{2+} solution	10.69	~10	0.5	25.78	25.02
			0.67	27.45	16.02
			0.83	30.12	11.21
			1	38.35	5.38
			4	50.12	5.65
			6	72.12	5.36
			24	83.58	4.10
			36	122.21	1.85
**Experiment 2**					
	**Weight (g)**	**Volume (mL)**	**Time (hours)**	**Ca^{2+} Conc. (ppb)**	**Pb^{2+} Conc. (ppb)**
CaS Aerogel	10.55		0	0	107.56
			0.17	2.21	66.98
			0.33	5.17	34.72
Pb^{2+} solution	10.15	~10	0.5	24.78	22.12
			0.67	26.51	15.78
			0.83	29.78	12.17
			1	39.39	4.45
			4	51.2	4.21
			6	75.48	4.33
			24	90.74	4.08
			36	125.27	2.85
**Experiment 3**					
	**Weight (g)**	**Volume (mL)**	**Time (hours)**	**Ca^{2+} Conc. (ppb)**	**Pb^{2+} Conc. (ppb)**
CaS Aerogel	10.48		0	0	99.16
			0.17	2.03	49.79
			0.33	7.17	26.48
Pb^{2+} solution	10.12	~10	0.5	21.81	24.21
			0.67	25.51	15.17
			0.83	31.27	10.23
			1	40.21	5.78
			4	49.29	5.13
			6	80.02	5.03
			24	92.81	2.51
			36	122.21	1.91

Calculation S1.1: Calculation of mean and standard deviation of Ca^{2+} concentrations (100 ppb) at different time intervals

Time Intervals (hours)	Experiment 1 (ppb)	Experiment 2 (ppb)	Experiment 3 (ppb)	Mean (ppb)	Standard Deviation (±ppb)
0	0	0	0	0	0
0.17	2.53	2.21	2.03	2.21	0.25
0.33	5.61	5.17	7.17	5.61	1.05
0.5	25.78	24.78	21.81	24.78	2.06
0.67	27.45	26.51	25.51	26.51	0.97
0.83	30.12	29.78	31.27	30.12	0.78
1	38.35	39.39	40.21	39.39	0.93
4	50.12	51.2	49.29	50.12	0.95
6	72.12	75.48	80.02	75.48	3.96
24	83.58	90.74	92.81	90.74	4.84
36	122.21	125.27	122.21	122.21	1.76

Calculation S1.2: Calculation of mean and standard deviation of Pb^{2+} concentrations (100 ppb) at different time intervals

Time Intervals (hours)	Experiment 1 (ppb)	Experiment 2 (ppb)	Experiment 3 (ppb)	Mean (ppb)	Standard Deviation (±ppb)
0	100.56	107.56	99.16	100.56	4.50
0.17	55.79	66.98	49.79	55.79	8.72
0.33	39.69	34.72	26.48	34.72	6.67
0.5	25.023	22.12	24.21	24.21	1.49
0.67	16.021	15.78	15.17	15.78	0.43
0.83	11.213	12.17	10.23	11.213	0.97
1	5.388	4.45	5.78	5.388	0.68
4	5.654	4.21	5.13	5.13	0.73
6	5.363	4.33	5.03	5.03	0.52
24	4.105	4.08	2.51	4.08	0.91
36	1.85	2.85	1.91	1.91	0.56

Calculation S1.3.1: Removal of 100 ppb Ca^{2+} from 10.46±0.17 g (~10 mL) of solution with 10.34±0.12 mg of CaS xerogel

Time Intervals (hours)	Mean (ppb)	Standard Deviation (±ppb)
0	0	0
1	24.064	0.93217
4	42.724	0.95772
6	35.528	3.96466
24	33.516	4.84327
36	17.376	1.76669

Calculation S1.3.2: Removal of 100 ppb Pb^{2+} from 10.46±0.17 g (~10 mL) of solution with 10.34±0.12 mg of CaS xerogel

Time Intervals (hours)	Mean (ppb)	Standard Deviation (±ppb)
0	113.56	7.50037
1	55.992	0.68342
4	59.524	0.73099
6	60.512	0.52725
24	60.236	0.91374
36	60.956	0.56083

Calculation S2: Removal of 20 ppm Pb^{2+} from 10.44 ± 0.45 g (~10 ml) of solution with 10.28±0.13 mg of CaS gel

Experiment 1					
	Weight (g)	Volume (mL)	Time (hours)	Ca^{2+} Conc. (ppm)	Pb^{2+} Conc. (ppm)
CaS Aerogel	10.38		0	1.29	18.83
			1	2.721	15.132
			3	8.916	12.64
Pb^{2+} solution	10.12	~10	6	17.026	2.372
			24	20.242	0.689
			36	29.49	0.21

Experiment 2					
	**Weight (g)**	**Volume (mL)**	**Time (hours)**	**Ca^{2+} Conc. (ppb)**	**Pb^{2+} Conc. (ppb)**
CaS Aerogel	10.19		0	0	20.31
			1	2.21	16.12
			3	5.17	10.76
Pb^{2+} solution	10.75	~10	6	24.78	4.39
			24	26.51	0.71
			36	29.78	0.51

Calculation S2.1: Calculation of mean and standard deviation of Ca^{2+} concentrations (20 ppm) at different time intervals

Time Intervals (hour)	Experiment 1 (ppm)	Experiment 2 (ppm)	Mean (ppm)	Standard Deviation (±ppm)
0	1.29	1.03	1.16	0.18
1	2.721	3.14	2.9305	3.04
3	8.916	9.27	9.093	0.25
6	17.026	18.78	17.903	1.24
24	20.242	25.75	22.996	3.89
36	29.49	26.91	28.2	1.82

Calculation S2.2: Calculation of mean and standard deviation of Pb^{2+} concentrations (20 ppm) at different time intervals

Time Intervals (hour)	Experiment 1 (ppm)	Experiment 2 (ppm)	Mean (ppm)	Standard Deviation (±ppm)
0	18.83	20.31	19.57	1.05
1	15.132	16.12	15.626	0.69
3	12.64	10.76	11.7	1.33
6	2.372	4.39	3.381	1.43
24	0.689	0.71	0.6995	0.02
36	0.21	0.51	0.36	0.21

Calculation S3: Removal of 200 ppm Pb^{2+} from 10.44 ± 0.45 g (~10 ml) of solution with 10.28±0.13 mg of CaS gel

Experiment 1					
	**Weight (g)**	**Volume (mL)**	**Time (hours)**	**Ca^{2+} Conc. (ppm)**	**Pb^{2+} Conc. (ppm)**
CaS Aerogel	10.38		0	1.03	201.11
			1	75.71	127.96
			3	139.47	101.62
Pb^{2+} solution	10.12	~10	6	203.12	66.79
			24	245.78	17.03
			36	275.45	4.74
**Experiment 2**					
	**Weight (g)**	**Volume (mL)**	**Time (hours)**	**Ca^{2+} Conc. (ppb)**	**Pb^{2+} Conc. (ppb)**
CaS Aerogel	10.19		0	0.33	194.17
			1	90.12	117.61
			3	131.21	90.26
Pb^{2+} solution	10.75	~10	6	194.29	49.94
			24	239.81	10.03
			36	259.59	5.44

Calculation S3.1: Calculation of mean and standard deviation of Ca^{2+} concentrations (200 ppm) at different time intervals

Time Intervals (hour)	Experiment 1 (ppm)	Experiment 2 (ppm)	Mean (ppm)	Standard Deviation (±ppm)
0	1.03	0.39	0.71	0.45
1	75.71	90.12	82.915	10.19
3	139.47	131.21	135.34	5.84
6	203.12	194.29	198.705	6.24
24	245.78	239.81	242.795	4.22
36	275.45	259.59	267.52	11.21

Calculation S3.2: Calculation of mean and standard deviation of Pb^{2+} concentrations (200 ppm) at different time intervals

Time Intervals (hour)	Experiment 1 (ppm)	Experiment 2 (ppm)	Mean (ppm)	Standard Deviation (±ppm)
0	201.11	194.17	197.64	4.91
1	127.96	117.61	122.785	7.32
3	101.62	90.26	95.94	8.03
6	66.79	49.94	58.365	11.91
24	17.03	10.03	13.53	4.95
36	4.74	5.44	5.09	0.49

Calculation S4: Removal of 2000 ppm Pb^{2+} from 10.44 ± 0.45 g (10 ml of solution with 10.28±0.13 mg of CaS gel

**Experiment 1**					
	**Weight (g)**	**Volume**	**Time (hours)**	**Ca^{2+} Conc. (ppb)**	**Pb^{2+} Conc. (ppb)**
CaS Aerogel	10.38		0	0.29	1955.9
			1	373.28	1242.7
			3	1475.97	128.07
Pb^{2+} solution	10.12	10	6	1595.84	125.27
			24	1914.68	103.27
			36	2251.16	93.27
**Experiment 2**					
	**Weight (g)**	**Volume (mL)**	**Time (hours)**	**Ca^{2+} Conc. (ppb)**	**Pb^{2+} Conc. (ppb)**
CaS Aerogel	10.19		0	0.45	2012.19
			1	342.12	1189.07
			3	1398.27	159.78
Pb^{2+} solution	10.75	10	6	1505.41	145.76
			24	1804.89	113.79
			36	2051.23	95.27

Calculation S4.1: Calculation of mean and standard deviation of Ca^{2+} concentrations (2000 ppm) at different time intervals

Time Intervals (hour)	Experiment 1 (ppm)	Experiment 2 (ppm)	Mean (ppm)	Standard Deviation (±ppm)
0	0.29	0.45	0.37	0.11
1	373.28	342.12	357.7	22.03
3	1475.97	1398.27	1437.12	54.94
6	1595.84	1505.41	1550.625	63.94
24	1914.68	1804.89	1859.785	77.63
36	2251.16	2051.23	2151.195	141.37

Calculation S4.2: Calculation of mean and standard deviation of Pb^{2+} concentrations (2000 ppm) at different time intervals

Time Intervals (hour)	Experiment 1 (ppm)	Experiment 2 (ppm)	Mean (ppm)	Standard Deviation (±ppm)
0	1955.9	2012.19	1984.045	39.80
1	1242.7	1189.07	1215.885	37.92
3	128.07	159.78	143.925	22.42
6	125.27	145.76	135.515	14.48
24	103.27	113.79	108.53	7.44
36	93.27	95.27	94.27	1.41

Calculation S5: Removal of 20000 ppm Pb^{2+} from 10.44 ± 0.45 g (10 ml) of solution with 10.28±0.13 mg of CaS gel

Experiment 1					
	**Weight (g)**	**Volume (mL)**	**Time Intervals (hours)**	**Ca^{2+} Conc. (ppb)**	**Pb^{2+} Conc. (ppb)**
CaS Aerogel	10.38		0	0.39	19878.12
			1	1494.7	18127.37
			3	2831.7	17687.32
Pb^{2+} solution	10.12	~10	6	3157.6	17327.11
			24	3062.1	17301.29
			36	3016.8	16327.03
**Experiment 2**					
	**Weight (g)**	**Volume (mL)**	**Time (hours)**	**Ca^{2+} Conc. (ppb)**	**Pb^{2+} Conc. (ppb)**
CaS Aerogel	10.19		0	0.47	20120.19
			1	1342.12	17891.07
			3	2398.27	17059.78
Pb^{2+} solution	10.75	~10	6	3005.41	17045.76
			24	3104.89	16913.79
			36	3251.23	16695.27

Calculation S5.1: Calculation of mean and standard deviation of Ca^{2+} concentrations (20000 ppm) at different time intervals

Time Intervals (hour)	Experiment 1 (ppm)	Experiment 2 (ppm)	Mean (ppm)	Standard Deviation (±ppm)
0	0.39	0.47	0.43	0.06
1	1494.7	1342.12	1418.41	107.89
3	2831.7	2398.27	2614.985	306.48
6	3157.6	3005.41	3081.505	107.61
24	3062.1	3104.89	3083.495	30.26
36	3016.8	3251.23	3134.015	165.77

Calculation S5.2: Calculation of mean and standard deviation of Pb^{2+} concentrations (20000 ppm) at different time intervals

Time Intervals (hour)	Experiment 1 (ppm)	Experiment 2 (ppm)	Mean (ppm)	Standard Deviation (±ppm)
0	19878.12	20120.19	19999.155	171.17
1	18127.37	17891.07	18009.22	167.09
3	17687.32	17059.78	17373.55	443.74
6	17327.11	17045.76	17186.435	198.94
24	17301.29	16913.79	17107.54	274.01
36	16327.03	16695.27	16511.15	260.39

Calculation S6: Weight percentages of CaS and CaO calculated from TGA data in **Figure 3**.

Initial weight of CaS FTP gel is 5.22 mg

Weight loss by water and residual ligands (Stages 1-3) is (100.02-87.5287) = 12.67%

Weight loss (Stages 1-3) is (12.672*5.22)/100 = 0.661 mg

Weight loss of CO_2 by the decomposition of $CaCO_3$ (Stage 4) is (87.528 – 67.9807) = 19.54%

Weight loss by CO_2 removal is (19.54 x 5.22)/100 = 1.02 mg

Weight of $CaCO_3$ = (100.09/44.01) x 1.02 = 2.32 mg

Weight of CaO = (2.32 x 56.08)/100.09 = 1.30 mg

Weight of CaS is (5.22 - 1.02 - 0.661 - 1.30) = 2.24 mg

Weight percentage of CaS is (2.24 x 100)/ (2.24 + 1.30) = **63.3%**

Weight percentage of CaO is (1.30 x 100)/ (2.24 + 1.30) = **36.7%**

Weight percentage of $CaCO_3$ in the original aerogel is (2.32/(2.32 + 2.24)) **= 50.1%**

Weight percentage of CaS in the original aerogel is (2.24/(2.32 +2.24)) = **49.9 %**

Calculation S7: Weight percentages of CaS and CaO calculated from TGA data in **Figure S9**.

Water + FTP loss = 3.9%

$$\text{Loss} = \frac{3.9 \times 19.86}{100} = 0.77454\ mg$$

Organics loss = 14%

$$\text{Loss} = \frac{14 \times 19.86}{100} = 2.78040\ mg$$

CO_2 loss from $CaCO_3$ decomposition = 6%

$$m_{\mathrm{CO_2}} = \frac{6 \times 19.86}{100} = 1.19160\ mg$$

Converted $CO_2 \rightarrow CaCO_3$

$$m_{\mathrm{CaCO_3}} = 1.19160 \times \frac{100.09}{44.01} = 2.71170\ mg$$

Converted $CaCO_3 \rightarrow CaO$

$$m_{\mathrm{CaO}} = 2.71170 \times \frac{56.08}{100.09} = 1.51870\ mg$$

Weight of CaS

$$m_{\mathrm{CaS}} = 19.86 - 0.77454 - 2.78040 - 1.51870 = 14.78636\ mg$$

Weight % of CaS and CaO (basis = CaS + CaO)

Total = $14.78636 + 1.51870 = 16.30506$mg

$$\%\ \mathrm{CaS} = \frac{14.78636}{16.30506} \times 100 = 90.68\%$$

$$\%\ \mathrm{CaO} = \frac{1.51870}{16.30506} \times 100 = 9.32\%$$

Weight % of $CaCO_3$ (basis = CaS + $CaCO_3$)

$$\%\ \mathrm{CaCO_3} = \frac{2.71170}{14.78636 + 2.71170} \times 100 = 15.50\%$$

Calculation S8: Theoretical exchange value of Pb^{2+} ion of CaS aerogel and maximum capacity

Theoretical Exchange Calculation for 1 gm of CaS gel where 49.1 wt% is CaS and 50.9 wt% of $CaCO_3$ according to TGA Data (Calculation S6)

1. Molar masses

- $M_{\mathrm{CaCO_3}} = 100.09$ g/mol
- $M_{\mathrm{CaS}} = 72.143$ g/mol

2. Convert wt.% to grams per 1 g of CaS aerogel

- Mass of $CaCO_3$:

$$m_{\mathrm{CaCO_3}} = 0.509 \text{ g}$$

- Mass of CaS:

$$m_{\mathrm{CaS}} = 0.491 \text{ g}$$

3. Convert each to moles

- Moles of $CaCO_3$

$$n_{\mathrm{CaCO_3}} = \frac{0.509}{100.09} = 0.005086 \text{ mol}$$

- Moles of CaS

$$n_{\mathrm{CaS}} = \frac{0.491}{72.143} = 0.006806 \text{ mol}$$

4. Total moles of Ca available to exchange with Pb^{2+}

Since 1 mol $CaCO_3$ or CaS binds 1 mol Pb^{2+}:

$$\begin{aligned} n_{\mathrm{Pb^{2+}},\mathrm{capacity}} &= n_{\mathrm{CaCO_3}} + n_{\mathrm{CaS}} \\ &= 0.005086 + 0.006806 \\ &= 0.011892 \text{ mol/g} \end{aligned}$$

5. Convert to mmol/g

$$0.011892 \text{ mol/g} \times 1000 = \mathbf{11.89\ mmol/g}$$

Mass of Pb^{2+}: 11.892 mmol g^{-1} × 207.2 mg $mmol^{-1}$ ≈ **2464 mg Pb^{2+}/g**.

Theoretical Exchange Calculation for 1 gm of CaS gel where 84.50 wt% is CaS and 15.5 wt% of $CaCO_3$ according to TGA Data (Calculation S7)

1. Molar masses

- $M_{CaCO_3} = 100.09$ g/mol
- $M_{CaS} = 72.143$ g/mol

2. Convert wt.% to grams per 1 g of CaS aerogel

- Mass of $CaCO_3$:

$$m_{CaCO_3} = 0.155 \text{ g}$$

- Mass of CaS:

$$m_{CaS} = 0.845 \text{ g}$$

3. Convert each to moles

- Moles of $CaCO_3$

$$n_{CaCO_3} = \frac{0.155}{100.09} = 0.001549\text{mol}$$

- Moles of CaS

$$n_{CaS} = \frac{0.845}{72.143} = 0.01171 \text{ mol}$$

4. Total moles of Ca available to exchange with Pb^{2+}

Since 1 mol $CaCO_3$ or CaS binds 1 mol Pb^{2+}:

$$\begin{aligned} n_{Pb^{2+},\text{capacity}} &= n_{CaCO_3} + n_{CaS} \\ &= 0.001549 + 0.01171 \\ &= 0.01326 \text{ mol/g} \end{aligned}$$

5. Convert to mmol/g

$$0.01326 \text{ mol/g} \times 1000 = \mathbf{13.26\ mmol/g}$$

Mass of Pb^{2+}: 13.26 mmol g^{-1} × 207.2 mg $mmol^{-1}$ ≈ **2747 mg Pb^{2+}/g**.

Maximum Capacity (mmol/g) calculation for CaS gel with ~50% $CaCO_3$

Maximum capacity,

$$q_e = \frac{V}{m}(C_0 - C_f)$$

= **3538.88** mg/g

Where, V = 10 mL (10.12 g)
M = 10.38 mg
C_o = 19999.16 ppm
C_f = 16511.15 ppm

Maximum Capacity (mmol/g)

3538.88 mg Pb^{2+}/g=17.08 mmol Pb^{2+}/g

Maximum Capacity (mmol/g) calculation for CaS gel with ~15% $CaCO_3$

Maximum capacity,

$$q_e = \frac{V}{m}(C_0 - C_f)$$

= **4664** mg/g

Where, V = 10 mL (10.37 g)
M = 10.58 mg
C_o = 20778.17ppm
C_f = 16114.21 ppm

Maximum Capacity (mmol/g)

4664 mg Pb^{2+}/g= **22.51** mmol Pb^{2+}/g

Rietveld refinement data for CaS aerogel heated to 800 °C in flowing Ar (Figure 4).

CaO Phase: Cubic, space group: Fm-3m, a = b = c = 4.814799 Å, α = β = γ = 90, V = 111.618 Å^3, weight percentage (%) = **29.8**

Atom	Multiplicity	x	y	z	Fraction
Ca	4	0	0	0	1
S	4	0.5	0.5	0.5	1

CaS Phase: Cubic, space group: Fm-3m, a = b = c = 5.70094 Å, α = β = γ = 90, V = 185.285 Å^3 weight percentage (%) = **70.2**

Atom	Multiplicity	x	y	z	Fraction
Ca	4	0	0	0	1
O	4	0.5	0.5	0.5	1

Weighted profile R-factor, R_{wp} = 11.8%, Number of refinements = 20.
